# Machine learning applications in risk management: Trends and research agenda

**DOI:** 10.12688/f1000research.161993.1

**Published:** 2025-02-25

**Authors:** Alejandro Valencia-Arias, Jesus Alberto Jimenez Garcia, Erica Agudelo-Ceballos, Aarón José Alberto Oré León, Ezequiel Martínez Rojas, Julio Leyrer Henríquez, Diana Marleny Ramírez-Ramírez

**Affiliations:** 1 Escuela de Ingeniería Industrial, Universidad Senor de Sipan, Chiclayo, 14001, Peru; 2 Dirección de Planificación y Desarrollo Institucional, Universidad Senor de Sipan, Chiclayo, 14001, Peru; 3 Departamento de Ciencias Administrativas, Instituto Tecnologico Metropolitano, Medellín, 50010, Colombia; 4 Instituto de Investigación de Estudios de la Mujer, Universidad Ricardo Palma, Santiago de Surco, 15039, Peru; 5 Vicerrectoría de Investigación e Innovación, Universidad Arturo Prat, Iquique, Tarapacá Region, Chile; 6 Universidad Ricardo Palma, Lima, Peru; 7 Ciencias económicas y administrativas, Instituto Tecnologico Metropolitano, Medellín, 50010, Colombia

**Keywords:** Decision Making, Random Forest, Big Data, Deep Learning, Security

## Abstract

**Background:**

Currently, risk management is positioned as a key issue in industries, which is why machine learning technologies have been integrated for impact assessment, prevention, and decision making in different sectors. However, there are still important research gaps, so the aim is to investigate research trends related to the use of machine learning in risk management.

**Methods:**

A bibliometric analysis is proposed based on the PRISMA-2020 declaration in the Scopus and Web of Science databases.

**Results:**

The results show a growing interest in the use of machine learning for risk management in the scientific literature. China, South Korea and the United States lead the research. The thematic development reflects emerging topics such as urban trees and Covid-19. Key terms include random forest, SVM, and credit risk assessment, while others such as prediction, postpartum depression, big data, and security are considered emerging topics, reflecting the cross-cutting nature and applicability of the topic across different sectors of society. Deep learning and feature selection are also priorities for enhancing machine learning applications in risk management

**Conclusions:**

Machine learning in risk management has grown exponentially, shifting focus from stacking to urban trees and Covid-19. Key contributors, journals, and nations shape this evolving research landscape.

## Introduction

Risk management is a very important activity for various industries and economic sectors that seek to detect, evaluate, and reduce uncertainties that may have a negative impact on the achievement of organizational objectives. Over the years, the exponential growth of data and the complexity of risks have challenged traditional risk management methodologies, thus the adoption of automatic learning or machine learning techniques has been considered an excellent tool. to address challenges in risk management. ^1, 2^

Conversely, machine learning represents a crucial component of artificial intelligence, facilitating the analysis of copious quantities of data, the identification of patterns, and the discovery of hitherto unrecognized insights. This markedly enhances the capacity to prevent and make decisions in risk management. The application of machine learning in risk management within supply chains has been well documented. For example, ^3^ utilised a deep-learning-based dual-stage PLS-SEM-ANN analysis to improve supply chain agility and resilience. A novel risk assessment method driven by big data focused on supply chains in the area of economic promotion at airports has demonstrated how machine learning contributes to the efficiency and safety of the transportation and distribution of goods. ^1^ Moreover, machine learning’s capacity for prediction in the context of supply chain risk management is of paramount importance in enabling the proactive identification of potential disruptions and the subsequent adjustment of logistics strategies. This underscores the value of machine learning in enhancing operational continuity and decision-making processes. ^4^

Furthermore, machine learning has also become a significant tool in the field of medical risk management, offering the potential to enhance the safety and quality of healthcare. For example, the application of machine learning in the field of diabetic healthcare has enabled the development of more precise predictive models, thereby facilitating more informed clinical decision-making and the creation of personalized treatment plans. ^5^ Moreover, sophisticated risk assessment instruments that employ machine learning integrate an array of predictive models to furnish precise and adaptable assessments of perioperative risks associated with medical procedures, thus facilitating the formulation of bespoke interventions for patient safety. ^6^ Furthermore, machine learning has been employed to forecast the likelihood of inpatient falls by examining both intrinsic and extrinsic variables, thereby underscoring its far-reaching influence on the domain of healthcare risk management. ^7^

Notably, machine learning is also having a significant impact on risk management in the financial sector. The use of machine learning for risk assessment and management in financial portfolios with high-dimensional problems has provided a deeper and more efficient understanding of the risks associated with investments and portfolios. ^8^ Similarly, machine learning has proven to be a valuable tool in credit risk assessment, as it can leverage human experience and computational intelligence to improve predictive accuracy by combining expert knowledge with genetic algorithms in credit selection characteristics for credit risk assessment. ^9^

The application of machine learning to risk management has gained significant importance in various fields such as medicine and engineering. Advances in this area have allowed the development of more accurate and effective risk assessment tools, which implies more informed decision making with less margin of error; examples include the integration of machine learning-based predictions for perioperative risk management ^6^ and the identification of perineural invasion in head and neck squamous cell carcinoma. ^10^

The interpretation of the models is an essential aspect in areas where decisions can have guidelines for human life because the ability to understand and justify the results of the model is essential to gain the confidence of the professionals and patients involved. In the decision-making process, it is therefore novel to incorporate the interpretability of models for cardiovascular risk assessment using machine learning, which allows a better understanding of how inflammation biomarkers influence risk estimation. ^2^

Furthermore, in non-medical areas such as accident risk management, research on the application of machine learning has made significant progress in recent years, as the application of these techniques in accident prevention and mitigation can have a significant impact on industrial safety and the protection of people and the environment. However, there are still important gaps that justify conducting an exhaustive bibliometrics, one of the main gaps being the adaptation of machine learning models to specific contexts. ^11^

However, In Malaysia, an accident risk analysis study was conducted, which highlights the importance of continuing to study and address the challenges that still exist in the implementation of these technologies, while ensuring that they are ethical, fair models and based on high quality data in order to achieve a positive and significant impact on society, particular challenges were identified in the application of supervised techniques, where the need to develop approaches and models that take into account the cultural, regulatory and environmental characteristics of each context stands out, allowing for more effective and personalized risk management. ^11^

Another relevant research gap is the early detection of emerging risks in different settings, one of which is the prediction of help-seeking in women with symptoms of postpartum depression, which shows the potential of machine learning to detect emerging risks in the health field. ^12^ However, it is necessary to deepen this line of research and apply it to other fields, such as industrial safety, environment, or finance, in order to anticipate and prevent possible threats before they become critical situations.

Existing machine learning models and risk management strategies can improve decision making and risk mitigation in an integrated way, but it is imperative to conduct a deeper investigation to understand how to maximize the capabilities of both approaches and optimize their integration to address the complex challenges of risk management in different industries. As mentioned above, it is important to investigate the effective integration of machine learning techniques with traditional risk management approaches, as suggested. ^13^ Therefore, the objective of this study is to examine the research trends related to the use of machine learning in risk management, so that a research agenda can be developed between 2007 and 2023, which allows oriented materialization of future research. In addition, the following research questions are posed:

- What are the years when there has been more interest in machine learning in risk management?
- What is the growth rate of scientific articles on machine learning in risk management?
- What are the main research references on machine learning in risk management?
- What is the thematic evolution derived from the scientific production on machine learning in risk management?
- What are the main thematic clusters on machine learning in risk management?
- What are the growing and emerging keywords in the research field of machine learning in risk management?
- Which topics are positioned as protagonists for the design of a research agenda on machine learning in risk management?

This article is structured in such a way that it begins with a review of the literature considered relevant in the field of the use of machine learning in risk management. Subsequently, the methodological section details the study design and the procedures used to collect and analyze the data in order to answer the questions raised above. The results are presented in the results section, where relevant information and statistics are presented, and their implications are discussed in the final section of the article.

## Methods

To achieve the objective of this research, an exploratory methodology is proposed, based on secondary research sources, through the performance of a bibliometric analysis that allows an evaluation of the scientific literature, specifically in relation to the use of machine learning in risk management. For this purpose, the parameters, or protocols of the international declaration PRISMA-2020 ^14^ will be followed.

### Inclusion criteria

The inclusion criteria for this bibliometric study on machine learning in risk management focus on two main aspects in document titles and metadata. First, records must contain terms related to “risk management” and its synonyms, such as “risk control.” Second, documents combining “risk management” with “risk assessment” and “risk analysis” are included, as these concepts provide a comprehensive view of the subject.

The exclusion process involves three phases. In the first phase, records with indexing errors are discarded. The second phase eliminates documents without full text access, applicable mainly to systematic literature reviews, since bibliometric analyses rely on available metadata. Finally, the third phase removes conference proceedings and documents with incomplete indexing that are not relevant, ensuring the quality and relevance of selected documents. These rigorous criteria aim to yield reliable results regarding the application of machine learning in risk management. ^14^

### Sources of information

In conducting this bibliometric study on machine learning in risk management, the Scopus and Web of Science databases were selected as the primary sources of information. ^15^ These databases provide comprehensive coverage of scientific journals, conferences, and patents, ensuring accurate and reliable indexing of articles. The combination of these databases will yield a more comprehensive and representative overview of the scientific output on this topic.

### Search strategy

In order to carry out the bibliometric search on the application of machine learning in risk management in the Scopus and Web of Science databases, two specialized search equations were designed, corresponding to the defined inclusion criteria and the search characteristics of each database.

For the Scopus database, the search formula was structured as follows TITLE (“risk management” OR “risk assessment” OR “risk analysis”) AND TITLE (“machine learning” OR “artificial intelligence” OR “predictive modelling” OR “data mining”). This formula combines the terms related to risk management in the title of the documents using the OR operator and similarly groups the terms related to machine learning. This combination of terms in the title of the documents makes it possible to obtain an exhaustive and precise search on the application of machine learning to risk management in the Scopus database.

On the other hand, for the Web of Science database, the search equation was formulated in a similar way: TI=(“risk management” OR “risk assessment” OR “risk analysis”) AND TI=(“machine learning” OR “artificial intelligence” OR “predictive modelling” OR “data mining”). In this equation, synonyms related to risk management and terms related to machine learning are again grouped using the OR operator in the title of the documents. This search structure makes it possible to obtain relevant and up-to-date results in the Web of Science database.

These specialized search applications have been carefully designed to ensure that the bibliometrics fully and accurately capture the scientific literature related to the application of machine learning in risk management, thus contributing to the success and quality of this research.

### Data management

During the development of the bibliometric analysis on the use of machine learning in risk management, Microsoft Excel was used to extract, store and process information from various databases. In addition, both the free software VOSviewer and Microsoft Excel were used to visualize the bibliometric indicators obtained. ^16^ The combination of both tools made it possible to create representative and accurate graphs of the collected data, which facilitated the analysis and presentation of the research results.

### Selection process

The PRISMA 2020 statement underscores the necessity of employing an automated classifier in the study selection process and validating its performance to mitigate the risks of missed or incorrectly classified studies. ^14^ In this study on machine learning in risk management, researchers employed Microsoft Excel automation tools developed in-house to apply inclusion and exclusion criteria independently. This approach was designed to reduce the likelihood of overlooking pertinent studies or misclassification by aligning the results of different reviewers.

### Data collection process

This study used Microsoft Excel as an automated tool to facilitate the data collection and organization process. All study authors acted as reviewers and independently validated the data. In addition, a collective data confirmation process was carried out to ensure sufficient verification until absolute convergence of the results obtained was achieved.

### Data elements

In this bibliometric study on machine learning in risk management, comprehensive data searches were conducted to identify and collect all relevant articles. A specialized search application was developed for each database to ensure comprehensive inclusion. However, to ensure the coherence and relevance of the study, any missing or unclear information was excluded, as were texts deemed to be of no relevance. This approach ensured that the research was focused on pertinent data, thereby aligning it with the study’s stated purpose and scope.

### Assessment of the risk of bias in the studies

A substantial emphasis was placed on the evaluation of the potential for bias in the selected studies. All authors contributed to this assessment through the utilisation of an enhanced automated Microsoft Excel tool, thereby ensuring consistency and accuracy. This collaborative approach, coupled with a reliable tool, was employed with the objective of minimising the potential for bias, thereby enhancing the reliability and validity of the research outcomes.

### Impact measures

This bibliometric study on machine learning in risk management acknowledges the paucity of analysis of diverse impact measures, which are more frequently employed in primary research. In accordance with the nature of secondary research, the study employed measures such as the number of publications and citations to assess relevance and impact. Furthermore, the study employed a temporal analysis of keyword usage to identify emerging trends. The manipulation and analysis of the data were conducted using Microsoft Excel, while VOSviewer ( VOSviewer - Visualizing scientific landscapes) was employed to ascertain the thematic associations between the documents. This methodological approach afforded a comprehensive understanding of scientific production in this field, thereby enhancing the research findings based on secondary sources.

### Synthesis methods

The bibliometric indicators of quantity, quality, and structure, as outlined by, ^17^ were automatically applied using Microsoft Excel, thereby streamlining the analysis process. The automation facilitated efficient information processing and ensured consistency in the application of the indicators, thereby enhancing the quality and reliability of the bibliometric research on machine learning in risk management.

### Assessment of reporting bias

It is of paramount importance to assess the risk of bias, given the potential for gaps in the synthesis of results. Bias may result from the use of specific synonyms in thesauri, such as IEEE, which can influence the criteria for inclusion, search strategies, and data collection. Furthermore, the use of conference proceedings may result in the omission of pertinent information due to incomplete indexing and the exclusion of irrelevant materials. It is imperative to consider these factors in order to achieve a more accurate and comprehensive evaluation of the collected data.

### Evaluation of certainty

**Figure 1.**
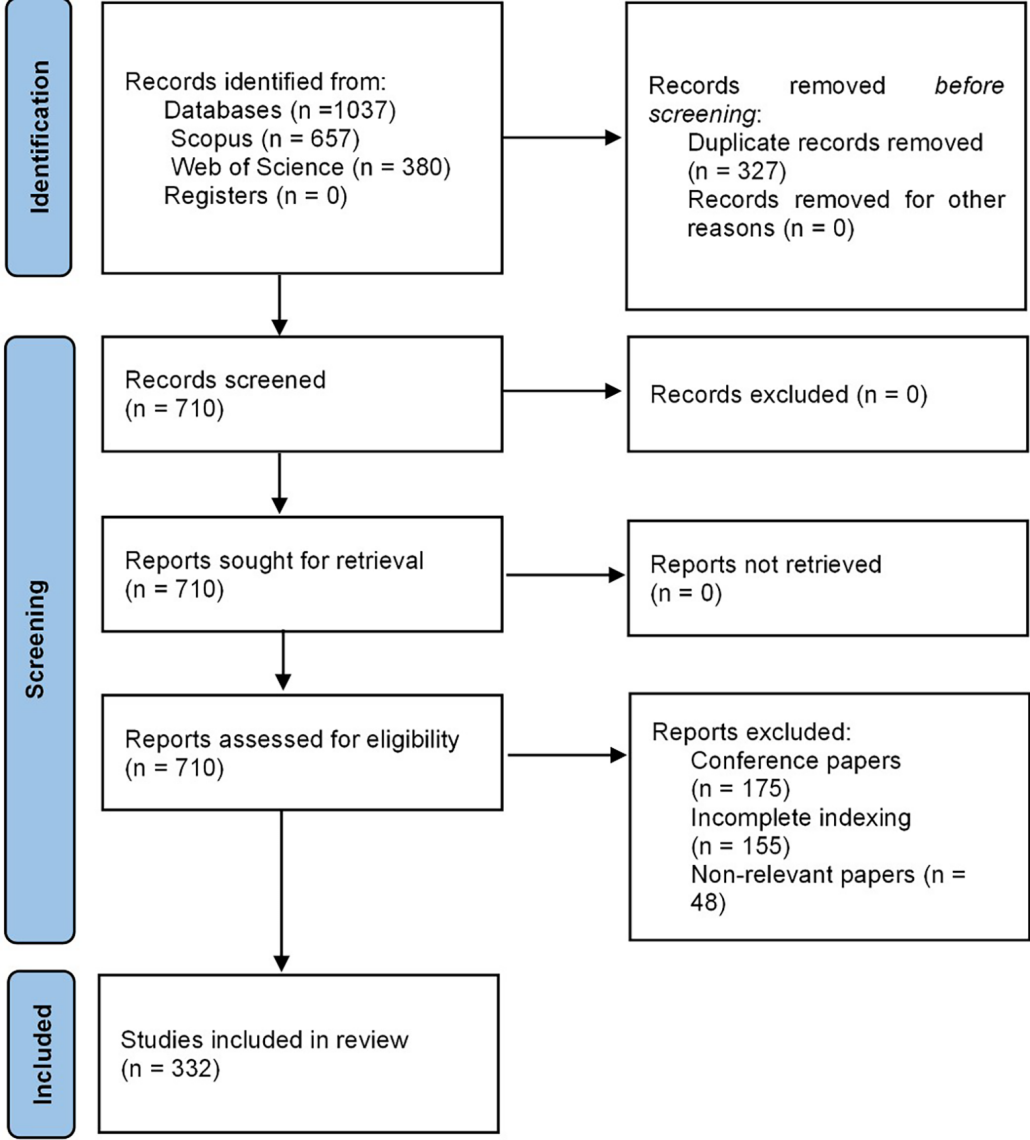
PRISMA flow chart. Own elaboration from Scopus and Web of Science.

Is comprehensive in its assessment of the certainty of the evidence. It considers a number of factors, including the independent application of inclusion and exclusion criteria, bibliometric indicators, and potential methodological biases. The discussion section addresses the limitations of the studies included in the review, thereby enhancing transparency. This comprehensive methodology is designed to yield a robust and reliable evaluation of the evidence on the topic. In the following Figure 1, the entire methodological design is evident from the PRISMA-2020 flow chart.

## Results

**Figure 2.**
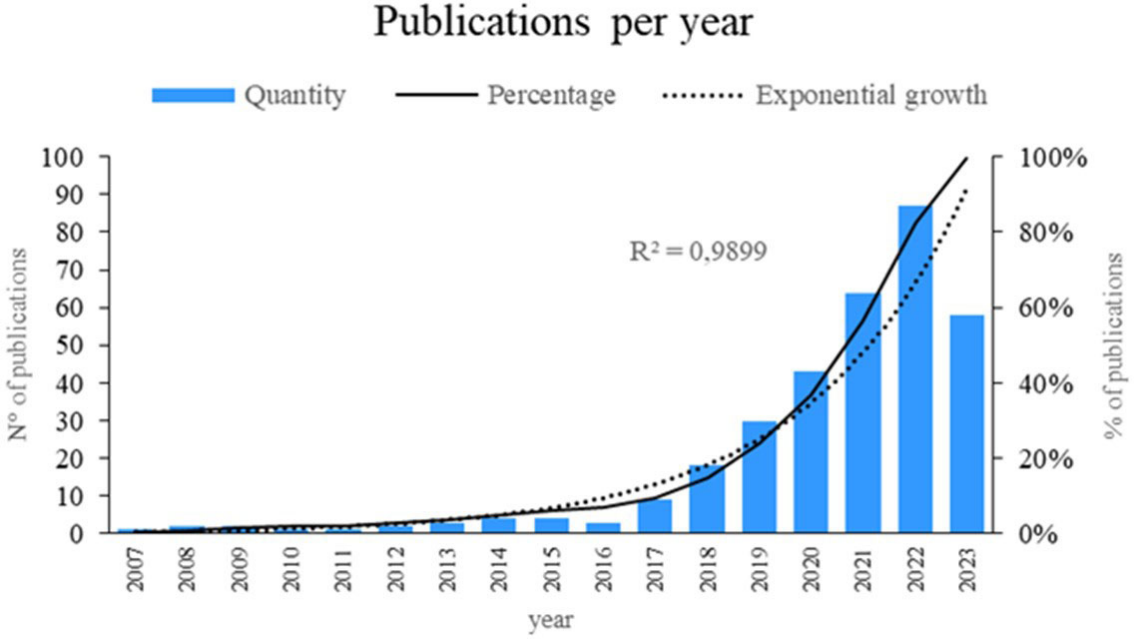
Publications by year. Own elaboration from Scopus and Web of Science.

Figure 2 presents a comprehensive analysis of the scientific literature in the field. The results indicated an exponential growth of 98.99% in published articles, representing a significant increase over time. The years 2023, 2022, 2021, and 2020 were the most notable in terms of publication output, reflecting a growing interest in the topic. These findings provide a clear and up-to-date perspective on the current state of the scientific landscape regarding the application of machine learning in risk management, thus contributing to the advancement of knowledge in this area.

**Figure 3.**
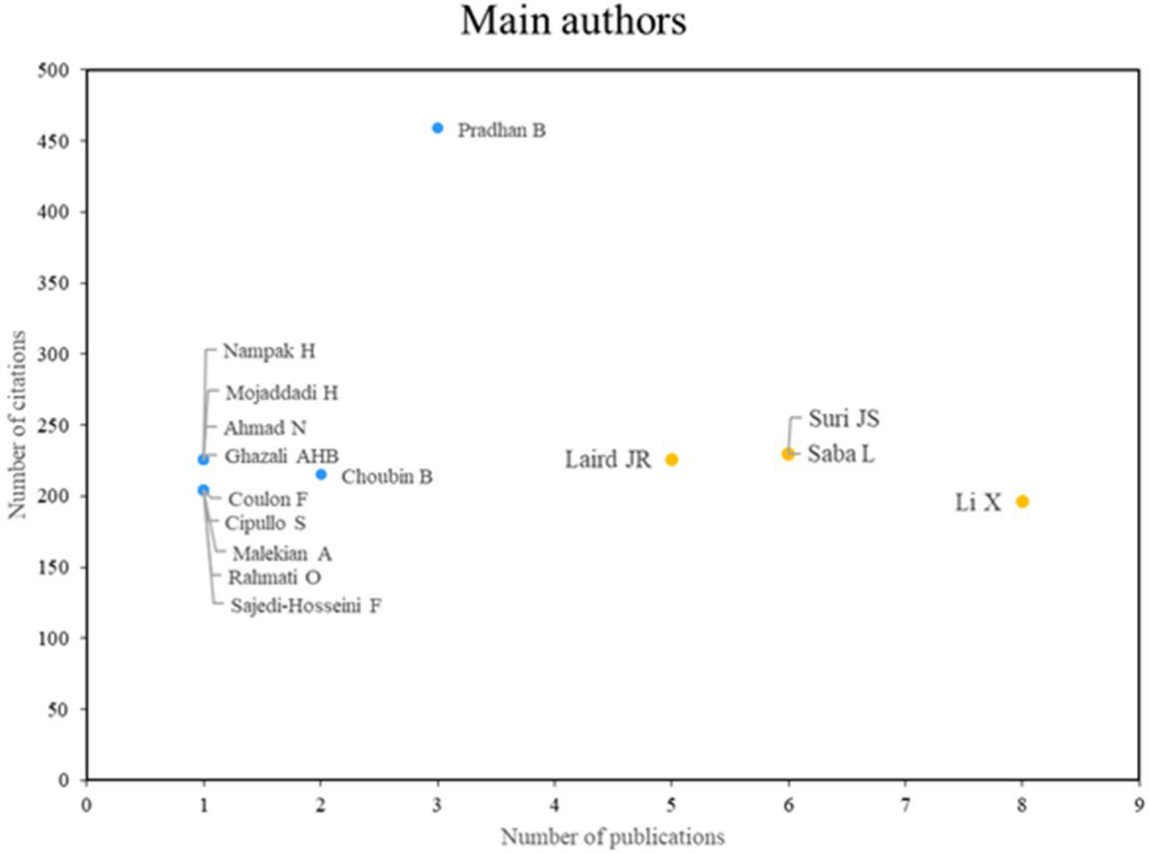
Main authors. Own elaboration from Scopus and Web of Science.

Figure 3 illustrates the two principal categories of distinguished authors. The first group, comprising Laird, Suri, Saba, and Li, is distinguished by high scientific productivity and a notable research impact, as evidenced by a substantial body of relevant publications. The second group, comprising researchers such as Pradhan and Choubin, is distinguished by their impactful contributions, despite exhibiting lower scientific productivity. Both groups demonstrate multifaceted contributions to the field.

**Figure 4.**
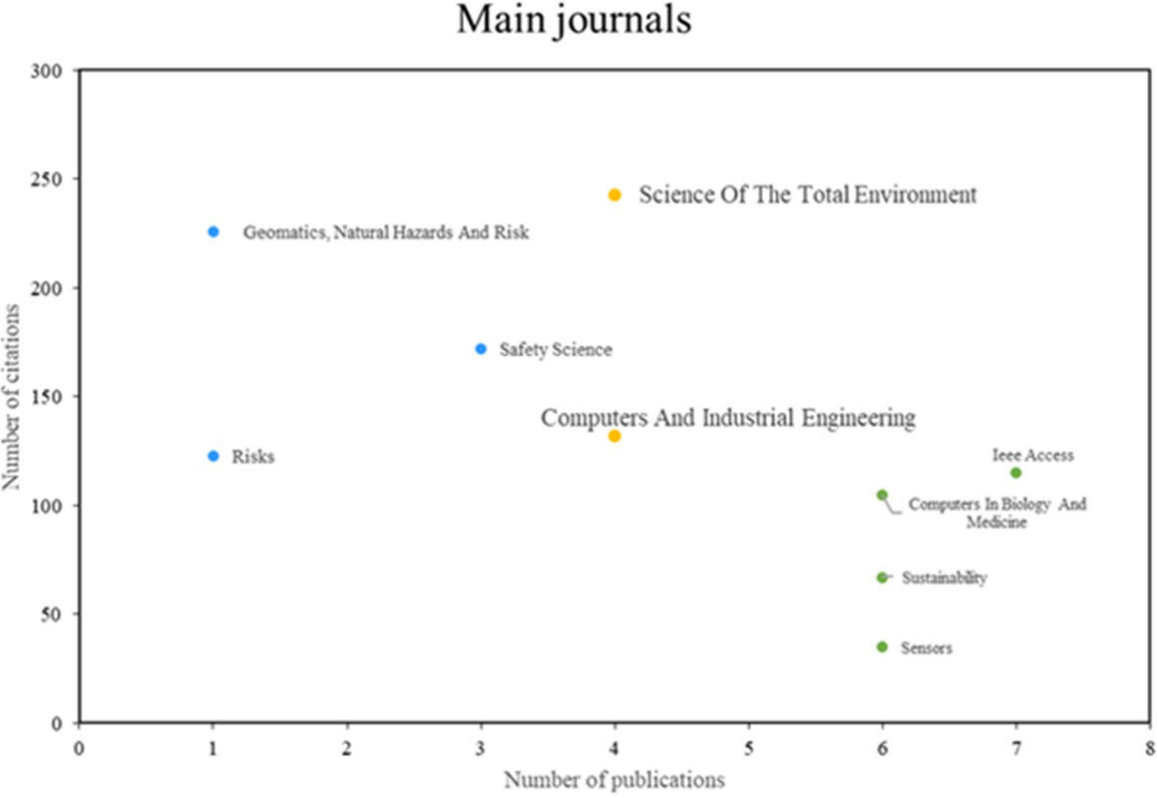
Main journals. Own elaboration from Scopus and Web of Science.

As illustrated in Figure 4, three principal categories of noteworthy journals were identified. The initial group comprises journals such as Science of the Total Environment and Computers and Industrial Engineering, which demonstrate remarkable productivity and impact, publishing a substantial number of pertinent articles and receiving a considerable number of citations. The second group includes journals such as Geomatics, Natural Hazards, and Risk and Safety Science, which are renowned for their high impact despite exhibiting lower productivity. Lastly, the third group comprises journals such as IEEE Access and Sensors, which are distinguished by high scientific productivity, although they may exhibit a lower number of citations compared to other journals.

**Figure 5.**
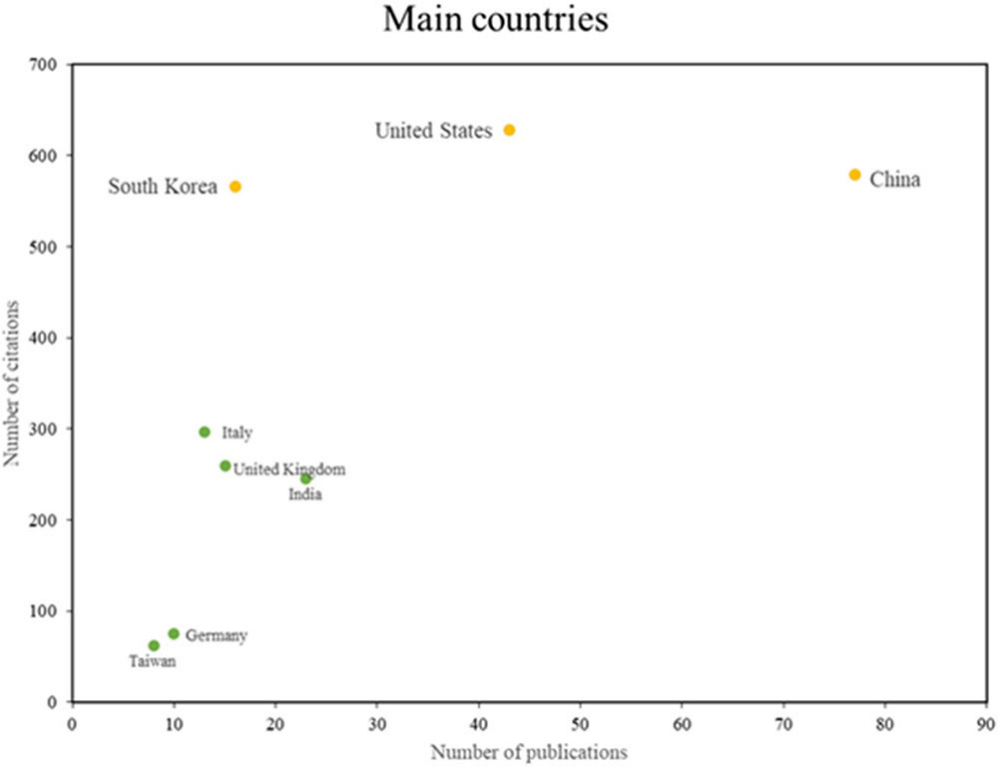
Main countries. Own elaboration from Scopus and Web of Science.

Figure 5 identifies two principal categories of countries that demonstrate particular excellence in this field. The initial group, which encompasses South Korea, the United States, and China, is distinguished by elevated levels of scientific productivity and impact, as evidenced by substantial research output and a multitude of citations. The second group, which includes countries such as Italy and India, exhibits robust scientific productivity but has not yet attained a comparable level of citations. This illustrates the diversity and global scope of research in this field, with different countries contributing distinctive strengths in productivity and impact.

**Figure 6.**
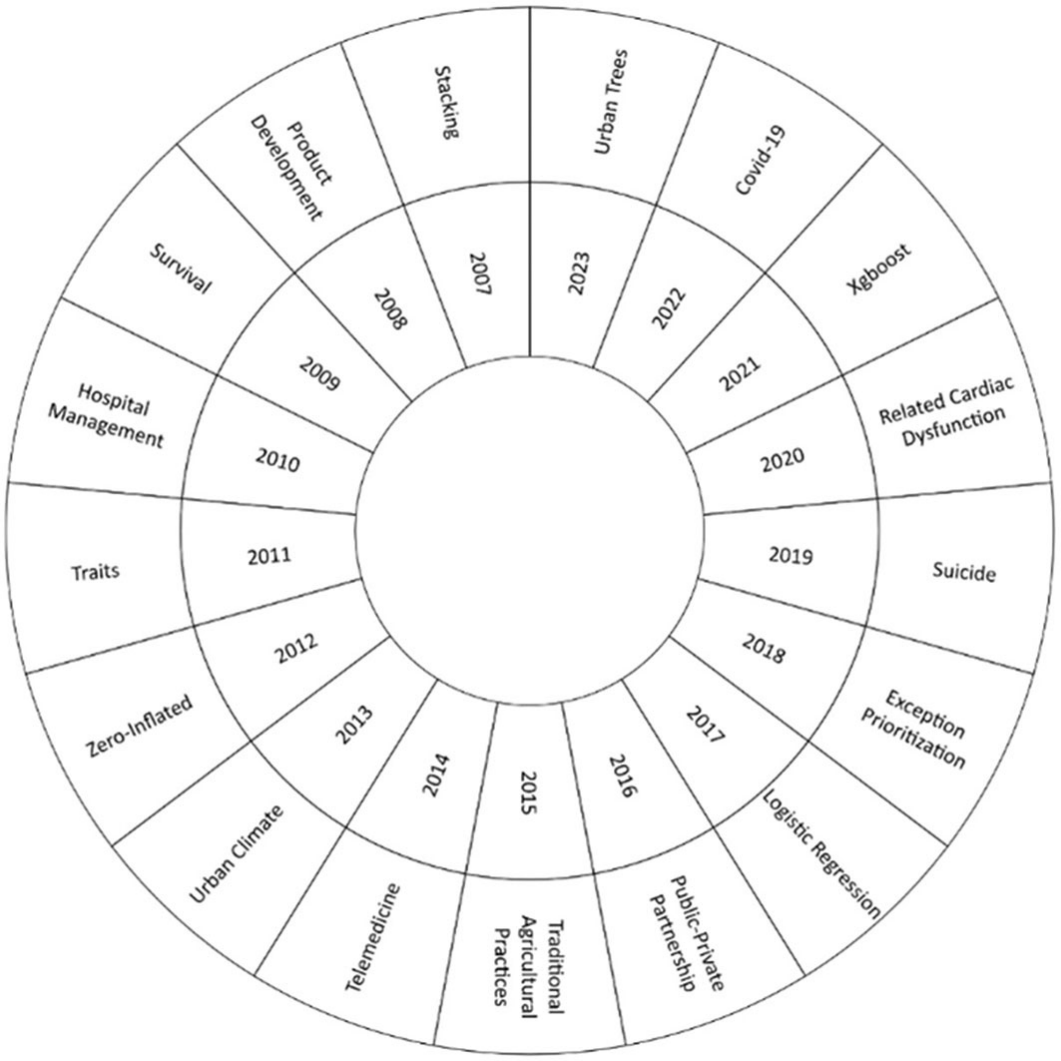
Thematic evolution. Own elaboration from Scopus and Web of Science.

In this bibliographic research, we examined how the machine learning approach to risk management has evolved over the years 2007 to 2023, as shown in Figure 6. The most frequent keyword in each year was analyzed to identify changes and trends in the field. At the beginning of the analysis, in 2007, the emergence of the term ‘stacking’ stood out. Over time, a significant thematic evolution was observed, with the emergence of relevant topics today, such as “Urban Trees”, “Covid-19”, “Xgboost”, “Related Cardiac Dysfunction” and “Suicide”.

**Figure 7.**
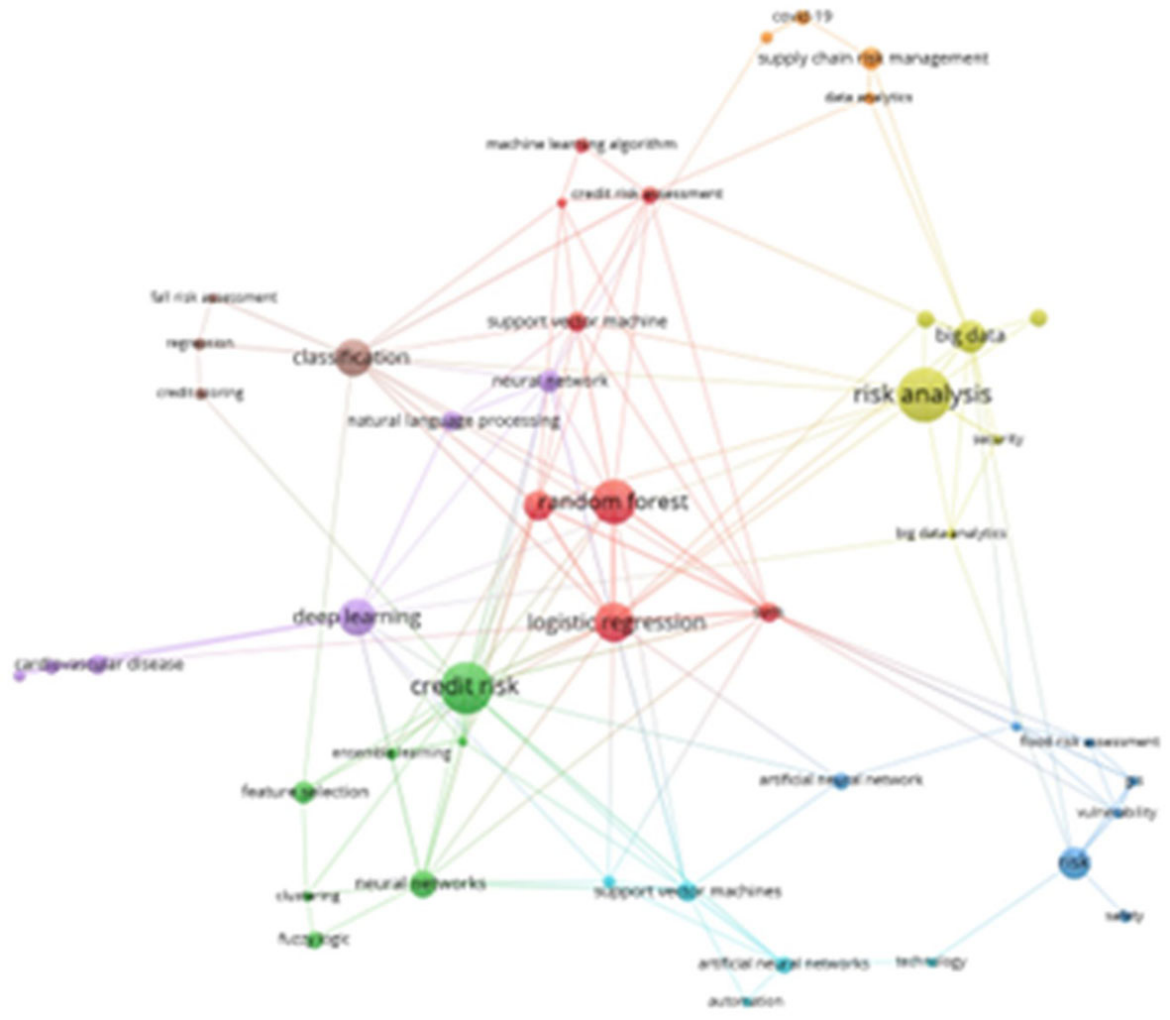
Keyword co-occurrence network. Own elaboration from Scopus and Web of Science.

The following presents an overview of a network of related keywords, which have been organized into eight thematic groups, as illustrated in Figure 7. The most prominent cluster, indicated in red, encompasses terms such as “Random Forest,” “Machine Learning Algorithm,” “Credit Risk Assessment,” “Support Vector Machine,” “Logistic Regression,” and “SVM.” The dark green cluster features terms such as “credit risk,” “ensemble learning,” “feature selection,” “neural networks,” “clustering,” and “fuzzy logic.” Additional clusters in lemon green, dark blue, light blue, purple, orange, and brown reflect various aspects of conceptual affinity within the field.

**Figure 8.**
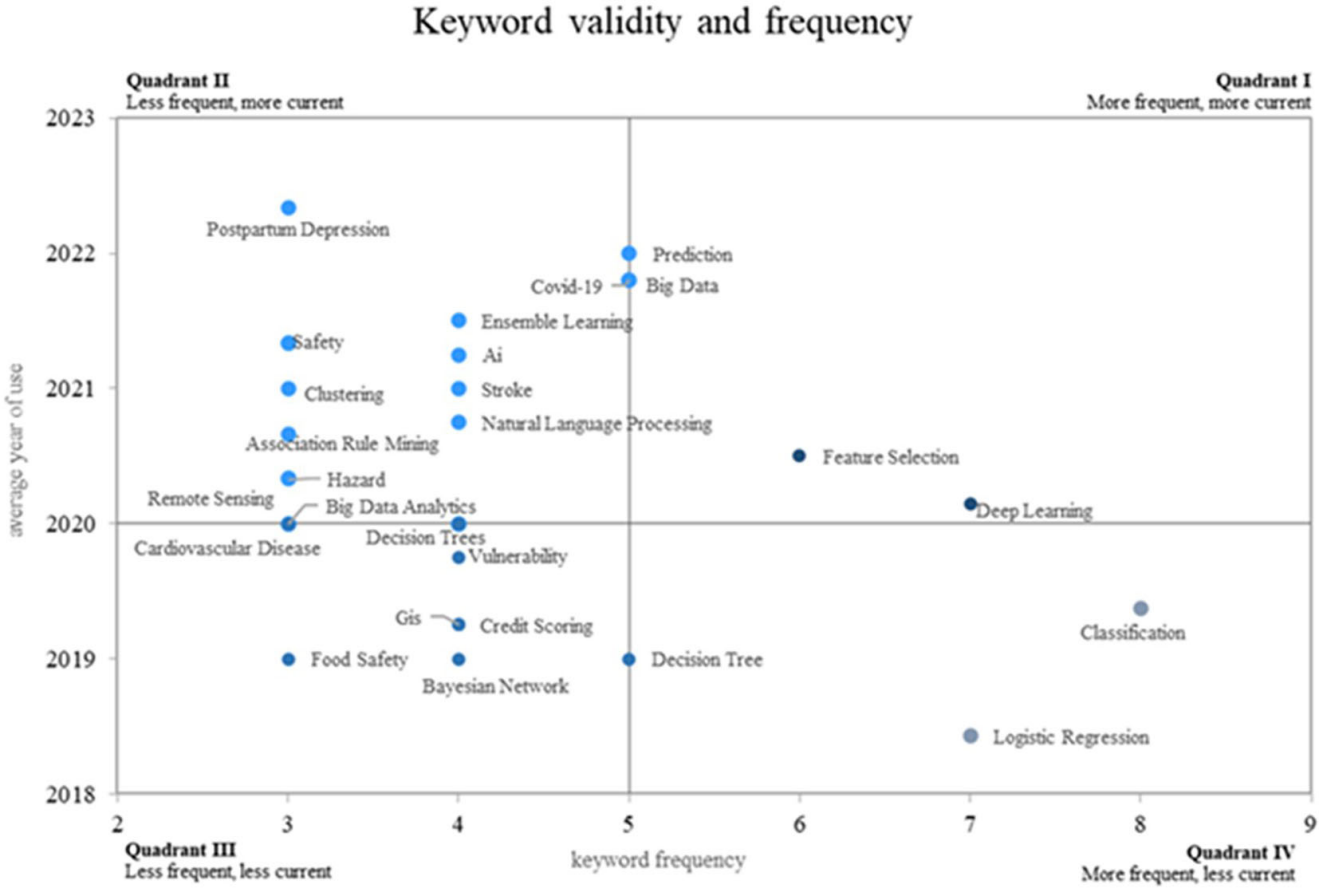
Validity and frequency of the keywords. Own elaboration from Scopus and Web of Science.

This research on the application of machine learning to risk management proposes a novel approach using a Cartesian plane that measures the frequency of use of keywords on the X-axis and the validity of use on the Y-axis, thus showing four different quadrants. as shown in Figure 8. Quadrant 4 contains descending concepts, including keywords such as classification, logistic regression, and decision tree. Quadrant 2 contains rare but highly topical words that are considered to be emerging, such as prediction, postpartum depression, Covid-19, big data and security. On the other hand, consolidated and growing terms such as prediction, big data, feature selection and deep learning are positioned in quadrant 1.

## Discussion

The discussion of the bibliometric analysis results presents an overview of the most salient findings, including annual scientific production, notable research references, the evolution of the subject, thematic clusters, and the frequency and validity of keywords. Furthermore, the section classifies fundamental keywords based on their function, examines the practical implications of such classification, discusses the limitations of the approach, and identifies research gaps. In conclusion, the paper puts forward a primary research agenda for the future, with a particular focus on machine learning in risk management.

### Analysis of the growth of scientific literature on the application of machine learning in risk management

A review of scientific output revealed a notable surge in publications pertaining to the deployment of machine learning in risk management between 2020 and 2023 ( Figure 2). For example, in 2020, a data mining-based framework for supply chain risk management was introduced. ^13^ Furthermore, an artificial intelligence approach for assessing the risk of infection with the novel coronavirus (2019-nCoV) in virtual medical visits was demonstrated. ^18^ In 2021, some authors investigated the potential of explanatory machine learning in credit risk management, ^19^ while others explored the use of hybrid artificial intelligence models for flood risk assessment in Quang Nam province, Vietnam. ^20^ These studies illustrate the accelerated evolution of machine learning applications across diverse domains, including supply chain management and public health.

In a continuation of this trend, a microbiological prediction model was developed for food risk analysis in 2022. This model is based on the Wiener process integrated with single-step kinetics. ^21^ Furthermore, deep learning was employed in the domain of supply chain risk management with the objective of enhancing agility, utilizing a dual PLS-SEM-ANN analysis. ^22^ By 2023, researchers had conducted a comprehensive study on the opportunities and challenges of supervised learning in maritime risk analysis. ^23^ Moreover, ^24^ conducted a literature review that underscored the significance of machine learning technology in bolstering supply chain risk management practices, proposing prospective avenues for future research to further integrate these sophisticated methodologies. The papers cited represent a sampling of the expanding body of research on the implementation of machine learning in risk management, addressing disparate topics and presenting innovative approaches that enhance the body of knowledge in this interdisciplinary field. Additionally, ^25^ conducted a comprehensive review of machine learning methods that have been specifically designed for engineering risk assessment. Their work illustrates the diverse applications and significance of machine learning in enhancing risk management frameworks across various industries.

### Analysis of research references on the application of machine learning in risk management

As for the main authors who stand out in terms of productivity and scientific impact in the application of machine learning in risk management, Laird, Suri and Saba, Li are shown in Figure 3. Laird, for his part, has excelled in research related to cardiovascular and cerebrovascular risk assessment using machine learning techniques; In a 2021 study, a multiclass machine learning approach to risk assessment of stroke and cardiovascular disease was presented, using predictors of carotid plaques with coronary angiography construct as the gold standard. ^26^ Also, Suri and Saba have contributed significantly in this field, as evidenced in. ^27^

For his part, author Li is considered a research benchmark in machine learning applications for risk assessment in process operations. In one of his studies, he presented a machine learning methodology for probabilistic risk assessment in gas leak incidents in underwater pipelines. ^28^ In addition, Li has worked on global flood risk assessment using machine learning models, applying several machine learning models to assess flood risk in global river basins. ^29^

On the other hand, Pradhan and Choubin stand out for their academic impact in research on risk assessment in natural disasters and water pollution, respectively. In a collaborative investigation, machine learning approaches were used to assess earthquake risk in Palu, Indonesia ^30^; similarly, they proposed a machine learning-based approach to assess the risk of nitrate contamination in groundwater ^31^; they also demonstrated the application of machine learning in urban flood risk assessment, integrating decision making and machine learning techniques. ^32^

Next, looking at the most prominent journals included in Figure 4, in terms of productivity and scientific impact, Science of The Total Environment and Computers & Industrial Engineering were found. The journal Science of The Total Environment has contributed significantly to the knowledge on this topic through research using machine learning approaches for earthquake risk assessment in Palu, Indonesia ^30^; machine learning has also been used for risk assessment of nitrate contamination in groundwater. ^31^

With regard to Computers & Industrial Engineering, another relevant journal that has made significant contributions to the field, a data mining-based framework for risk management in supply chains has been presented ^13^; in addition to a systematic review on the future of artificial intelligence and its impact on risk management in supply chains. ^33^

Similarly, journals such as Geomatics, Natural Hazards and Risk and Safety Science also stood out in terms of impact. A machine learning approach for flood risk assessment using remote sensing data and GIS was published in 2017. ^34^ In the field of mining safety, artificial intelligence was applied to assess gas risks in coal mines in 2921. ^35^

Finally, in the group of journals with high scientific productivity but low number of citations, IEEE Access and Sensors were found. Studies based on machine learning of the cryptocurrency market for financial risk management have been carried out ^36^; other investigations have studied the profiling of cybernetic attackers for risk analysis through machine learning. ^37^ These journals have been essential for the dissemination of innovative and relevant research in the field of machine learning applications in risk management.

In terms of the main countries, Figure 5 shows that South Korea, the United States and China stand out for their scientific production on the application of machine learning in risk management; these countries have made significant contributions in this area, demonstrating their leadership in the research and application of advanced machine learning techniques for risk management; In the case of South Korea, machine learning-based analyses have been conducted for financial risk management in the cryptocurrency market ^36^; similarly, in the field of sports medicine, machine learning models have been applied to analyze the risks of anterior cruciate ligament injuries. ^38^

Researchers in the United States worked on the development of risk assessment tools using machine learning; in 2023 they proposed an approach to integrate machine learning predictions into perioperative risk management ^6^ and in 2022 they developed machine learning models for individualized assessment of necrosis risk in mastectomy flaps. ^39^

In the same way, China is a benchmark in the application of automatic learning for risk management, studies have addressed risk assessment in supply chains using big data and machine learning, ^1^ in addition, they have proposed a machine learning-based method for pre-eclampsia risk assessment and related gene discovery. ^40^

Looking at Italy and India, although they are mainly recognized for their scientific productivity, they also have valuable research in risk management with the application of machine learning, in 2023 a study was conducted on defect detection using machine learning for asset risk management of existing bridges, ^41^ as well as in 2022 they focused on the use of deep learning and data analytics to improve agility in risk management in supply chains. ^22^

### Analysis of the thematic evolution of the application of machine learning in risk management

For the thematic evolution in Figure 6, it was found that the concept of stacking had a significant relevance in the first years of the application of machine learning in risk management. One of the relevant studies in this sense was “Credit risk analysis using a hybrid data mining model”. ^42^ This concept combines several machine learning models in order to improve the accuracy of predictions and allows to deal effectively with the assessment of credit risk. This methodology paved the way for the use of more advanced techniques in risk management and laid the foundations for the thematic evolution of the literature on the application of machine learning in risk management. Over the years, knowledge has expanded, and new approaches have been incorporated.

In the current state of the subject, key concepts that have received significant attention in recent literature have been identified, highlighting their relevance in different research areas, among which the concept of “suicide” has been the subject of study in 2019. It is approached from the perspective of suicide risk management in patients through the analysis of data from electronic health records, ^43^ which allows the development of clinical support strategies for decision-making in suicide prevention, demonstrating the usefulness and potential of machine learning techniques in the field of mental health.

In 2020, the concept of ‘Related Cardiac Dysfunction’ was highlighted, with an emphasis on the application of machine learning to assess the risk of cardiac dysfunction in patients undergoing cancer treatment. ^44^ The use of machine learning techniques has enabled a more accurate and personalized assessment of the risk of cardiac damage associated with cancer treatment, with a significant impact on the quality of life and survival of cancer patients.

By 2021, the focus will be on the concept of “Xgboost”, a machine learning technique that has gained popularity due to its ability to improve the accuracy of predictors. ^26^ This methodology has been applied in the context of cardiovascular risk and stroke, allowing a more effective and reliable assessment of risk by incorporating predictors based on carotid plaques and coronary angiography determination.

On the other hand, the year 2022 was strongly influenced by the concept of “Covid-19”, where the use of machine learning has stood out in the field of risk management related to the pandemic. ^18^ The development of approaches based on artificial intelligence has been essential to assess the risk of infection in virtual visits, which has been relevant in medicine and in the adoption of preventive measures in the fight against the spread of the virus.

Finally, in 2023, the concept of “urban trees” emerged as a topic of interest in risk management in urban areas, ^45^ the application of artificial intelligence has allowed the optimization of the associated risk assessment. to urban trees in localities in Brazil, facilitating the identification and prioritization of risk prevention and mitigation actions in urban environments.

### Analysis of thematic clusters on the application of machine learning in risk managemente

The bibliometric analysis facilitated the identification of disparate thematic clusters, as illustrated in Figure 7, which demonstrate the affinities between the most recurrent terms in the scientific literature. Of particular note is the red cluster, which is comprised of keywords such as “Random Forest,” “Machine Learning Algorithm,” “Credit Risk Assessment,” “Support Vector Machine,” “Logistic Regression,” and “SVM.” This cluster reflects a strong association between machine learning techniques, credit risk assessment, and specific algorithms utilized for credit analysis.

The second most significant cluster, depicted in dark green, was distinguished by keywords such as “credit risk,” “conjoint learning,” “feature selection,” “neural networks,” “clustering,” and “fuzzy logic.” This cluster demonstrates a correlation between concepts pertaining to credit risk assessment through the application of machine learning techniques and feature selection algorithms. An exemplary piece of research in this cluster is the 2021 study, which integrates expert insight with genetic algorithms to facilitate feature selection in credit risk assessment. ^9^ Moreover, the nascent cluster pertaining to digital assets underscores the paramount importance of optimizing portfolio management and risk assessment through the application of deep learning techniques. This is exemplified by the work of, ^46^ who concentrate on predictive analysis to augment decision-making processes in this rapidly evolving field.

### Analysis of frequency and conceptual validity around the use of machine learning in risk management

In the analysis of the Cartesian plane presented in Figure 8, quadrant 4 was identified as the one that contains decreasing or less used concepts compared to previous periods, in this quadrant are keywords such as “classification”, “logistic regression” and “decision tree”, these concepts, which in the past may have been more frequent in the scientific literature on the subject, show a decrease in their use in recent years.

For the concept of “classification”, relevant research was found that represents its past use, the authors applied a classification approach based on machine learning for the evaluation of spatiotemporal risks in crime data, ^47^ this concept may have been more prevalent in earlier studies related to data classification and analysis for risk management.

On the other hand, “logistic regression” has been used historically in financial risk management, as shown in, ^48^ but its presence seems to have decreased in recent studies on the topic. “Logistic regression" is a classic statistical method that has been widely used in various fields, but with the advancement of more complex machine learning techniques, its use in risk management may have decreased.

Finally, the concept of “Decision Tree”, which also shows a decreasing trend in recent years, has been applied in the optimization of risk analysis, as can be seen in, ^45^ where artificial intelligence is used for risk assessment in the context of managing trees in a specific region. While decision trees have been a valuable tool in risk management, other, more advanced approaches have gained popularity in recent years.

Quadrant 2 of the analysis of the Cartesian plane stood out for the grouping of emerging concepts in the scientific field of the application of machine learning in risk management; there are keywords such as “prediction”, “postpartum depression”, “Covid-19”, “big data” and “security”, which represent growing areas of great importance today and in the near future.

The concept of “prediction” has become fundamental in risk management, as it makes it possible to anticipate possible scenarios and assess the likelihood of future events, where ^47^ machine learning is used to perform conditional classification and assess spatio-temporal risks in crime data, the term “prediction” becomes relevant for informed decision-making and incident prevention.

For its part, ‘postpartum depression’ has also become a prominent research topic in risk management, as shown in, ^12^ where machine learning is applied to predict risk seeking. To help women with symptoms of postpartum depression, early detection and proper management of this condition is essential to minimize the risks associated with maternal mental health.

On the other hand, the “Covid-19” pandemic has led to a significant increase in risk studies and crisis management, as evidenced in, ^18^ where the issue of infection risk assessment using intelligence is addressed. During the virtual visit, the application of machine learning in the management of health-related risks was crucial to make informed decisions in the midst of a health crisis.

“Big data” has also gained importance in risk management, as shown in, ^1^ where a method based on massive data is proposed for risk assessment in supply chains. The ability to process and analyze large amounts of data has significantly improved decision making and the identification of potential risks in complex environments.

Finally, the term ‘safety’ highlights the concern for safety in various sectors, and in Ref. 11, the use of machine learning for accident risk analysis is reviewed, particularly in Malaysia. This approach is relevant to industrial risk management and occupational safety, with the aim of preventing incidents and improving safety in the work environment.

Quadrant 1 of the Cartesian plane analysis revealed growing, leading and consolidated concepts in the application of machine learning to risk management. Among the prominent keywords in this quadrant are “prediction”, “big data”, “feature selection” and “deep learning”, which play a fundamental role today and have great potential for the near future.

“Prediction” is one of the most solid and widely studied concepts in risk management, it refers to the ability of machine learning to make accurate and reliable predictions about future events, research papers such as ^47^ show how the use of machine learning algorithms has significantly improved the ability to prevent risks in various areas, such as the analysis of crime data in spatiotemporal environments.

“Big data” has also established itself as an important concept for risk management in various fields, some research shows how the analysis of large amounts of data has allowed the identification of complex patterns and trends in risk assessment in supply chains. ^1^ The effective use of “big data” provides a deeper and more complete vision of potential risks and facilitates more informed decision making.

"Feature Selection" is another integrated tool in risk management, and it has been shown that the use of feature selection techniques allows the identification of key variables that influence risk prediction, thus improving the accuracy and efficiency of risk models. In the case of machine learning, ^49^ this ability to select the most relevant features has a significant impact on the early identification and mitigation of risks in a variety of applications.

Finally, ‘deep learning’ has emerged as a powerful and promising approach to risk management, showing how the use of U-series architectures has revolutionized image analysis in stroke risk assessment, ^50^ ‘deep learning’ enables a deeper representation of data, which has led to significant advances in risk analysis and prediction in various fields such as medicine.

### Classification of keywords on the use of machine learning in risk management according to their function

Table 1 is a fundamental component of this bibliometric study, as it classifies emerging and expanding machine learning concepts in risk management through an analysis of recent scientific literature. The key terms are organized according to function, thereby providing a systematic overview of the most active and promising research areas and applications in this evolving field.

**Table 1.** Classification of keywords according to their function. Own elaboration from Scopus and Web of Science.

Keyword	Related tools	Applications	Features
Prediction	Regression models, neural networks, support vector machines	Predicting future trends	Event prediction based on historical data.
Postpartum Depression	Natural language processing, sentiment analysis, classification	Identifying and monitoring postpartum depression	Language analysis to detect signs of depression.
Covid-19	Data mining, machine learning, epidemiological models	Predicting the spread of disease	Data analysis to understand the pandemic.
Big data	Data Analytics, Data Visualization, Data Integration	Extracting valuable information from large amounts of data	Managing and analyzing large data sets.
Safety	Risk Assessment, Fault Detection, Incident Reporting	Improve safety in the workplace	Early failure detection and accident prevention.
Feature selection	Recursive Feature Elimination, Principal Component Analysis	Selecting the most relevant features	Reducing dimensionality and improving model performance.
Deep Learning	Convolutional Neural Networks, Recurrent Neural Networks	Detect complex patterns	Hierarchical learning and high level representations.

The previous classification, as can be seen, is based on new concepts such as prediction, postpartum depression, Covid-19, big data, security, feature selection and deep learning, this classification becomes an important element for future research to support its studies based on these keywords.

### Practical implications

This bibliometric analysis revealed a thematic evolution from an initial focus on stacking to a deeper analysis of concepts such as Urban Trees, Covid-19, Xgboost, Related Cardiac Dysfunction and Suicide, which have important practical implications in the field of risk management. These results show that the scientific community is adapting its views to address urgent and emerging risks associated with specific problems, such as the Covid-19 pandemic and mental disorders.

Likewise, the analysis of the keyword co-occurrence network provides valuable information on the conceptual affinity of relevant terms in risk management, the identification of key terms such as Random Forest, Machine Learning Difference, Credit Risk Assessment, Support Vector Machine, Logistic Regression and Svm in the main thematic cluster suggests the importance of these tools in decision-making related to financial and credit risk management, this information can be very useful for professionals and experts in the field when designing strategies and models to reduce risk in different contexts.

In addition, keyword frequency and currency analysis show a different view of emerging trends in risk management: the fact that concepts such as classification, logistic regression and decision tree are declining in relevance, while terms such as prediction, postpartum depression, covid-19, big data and safety are emerging, reflects the changing dynamics of the field and the need to address new challenges and emerging issues.

On the other hand, the growth of concepts such as Prediction, Big Data, Feature Selection and Deep Learning highlights the importance of applying advanced machine learning techniques in risk management. These tools provide the ability to analyze large amounts of data, select key features and make accurate predictions, which can significantly improve decision making in risk identification, assessment and mitigation in different sectors and environments.

### Limitations

Firstly, the selection of the databases used, such as Scopus and Web of Science, could have omitted some relevant publications in the field found in other sources not included in this study, which could have resulted in a partial view of the scientific production in the area studied.

Similarly, although tools such as Microsoft Excel and VOSviewer have been used to define bibliometric indicators of quantity, quality and structure, it is possible that these tools have not fully captured the complexity and diversity of the scientific literature in this field; some qualitative aspects of the publications, such as the depth of the analyses or the quality of the research methods used, may have been outside the scope of the quantitative indicators used.

It should also be noted that due to time and resource constraints, some relevant publications may have been excluded from the analysis, which may have affected the representativeness and completeness of the results presented.

### Research gaps

Table 2 is attached, which presents the main research gaps identified in this topic. This table objectively and neutrally describes the areas where more attention and development are needed to close the existing gaps in the effective use of machine learning in risk management.

**Table 2.** Research gaps. Own elaboration from Scopus and Web of Science.

Gap category	Gaps identified	Justification	Questions for future researchers
Thematic gaps	- Lack of studies that specifically address the application of machine learning to natural disaster risk management.	Current bibliometrics show extensive research in the field of risk management, but few studies focus on the use of machine learning for natural disasters, which could be of great benefit.	How can machine learning improve the prediction and mitigation of risks associated with natural disasters such as earthquakes, floods or hurricanes? What are the best practices for applying machine learning in this context?
	- Lack of focus on the application of machine learning for risk management in specific industries, such as aviation or energy.	Although progress has been made in general risk management, there is little research on how machine learning could be adapted and optimized for specific industries.	What are the unique challenges of risk management in specific industries and how can machine learning effectively address them? What are the opportunities for implementing machine learning models in the aviation or energy industries?
Geographical gaps	- Insufficient representation of studies from developing countries in the application of machine learning for risk management.	Most research focuses on developed countries, which limits the understanding of how machine learning can benefit developing countries.	What are the specific barriers to the adoption of machine learning in developing countries in terms of resources, technology and training? How can these barriers be overcome to encourage wider use of machine learning in risk management in these regions?
	- Lack of studies addressing specific risks related to climate change and their application with machine learning in vulnerable regions.	Given the increasing risks associated with climate change, it is crucial to explore how machine learning can contribute to mitigation and adaptation in vulnerable regions.	How can machine learning improve climate risk assessment and forecasting in regions prone to extreme events? What specific machine learning approaches are most effective in addressing climate change-related risks?
Interdisciplinary gaps	- Limited integration of multidisciplinary approaches in the application of machine learning for risk management.	Bibliometrics show a predominance of other research focused on computer science, which may miss opportunities for collaboration with other disciplines.	How can collaboration between machine learning experts, risk management experts and other disciplines (such as engineering, social sciences or ecology) enrich the development and implementation of machine learning solutions for risk management?
	- Lack of research that combines uncertainty analysis in the application of machine learning to risk management.	Uncertainty assessment is crucial for informed decision-making in risk management, and its integration with machine learning could improve the accuracy and reliability of results.	How can uncertainty analysis be incorporated into machine learning models used for risk management? What are the best strategies for quantifying and communicating uncertainty in machine learning-based decision making?
Temporary gaps	- Limited availability of research that assesses the long-term sustainability of machine learning solutions for risk management.	It is important to understand how machine learning applications evolve and are maintained over time, as risks and challenges may change over time.	What is the long-term impact of machine learning solutions on risk management? How can organizations ensure that machine learning applications are sustainable and remain relevant in an ever-changing environment?

In terms of coherence, a total of 4 research gaps have been characterized in terms of subject matter, geography, interdisciplinarity and temporality, which provide information on the various ways in which future studies can be considered to meet existing research and knowledge needs.

### Research agenda

**Figure 9.**
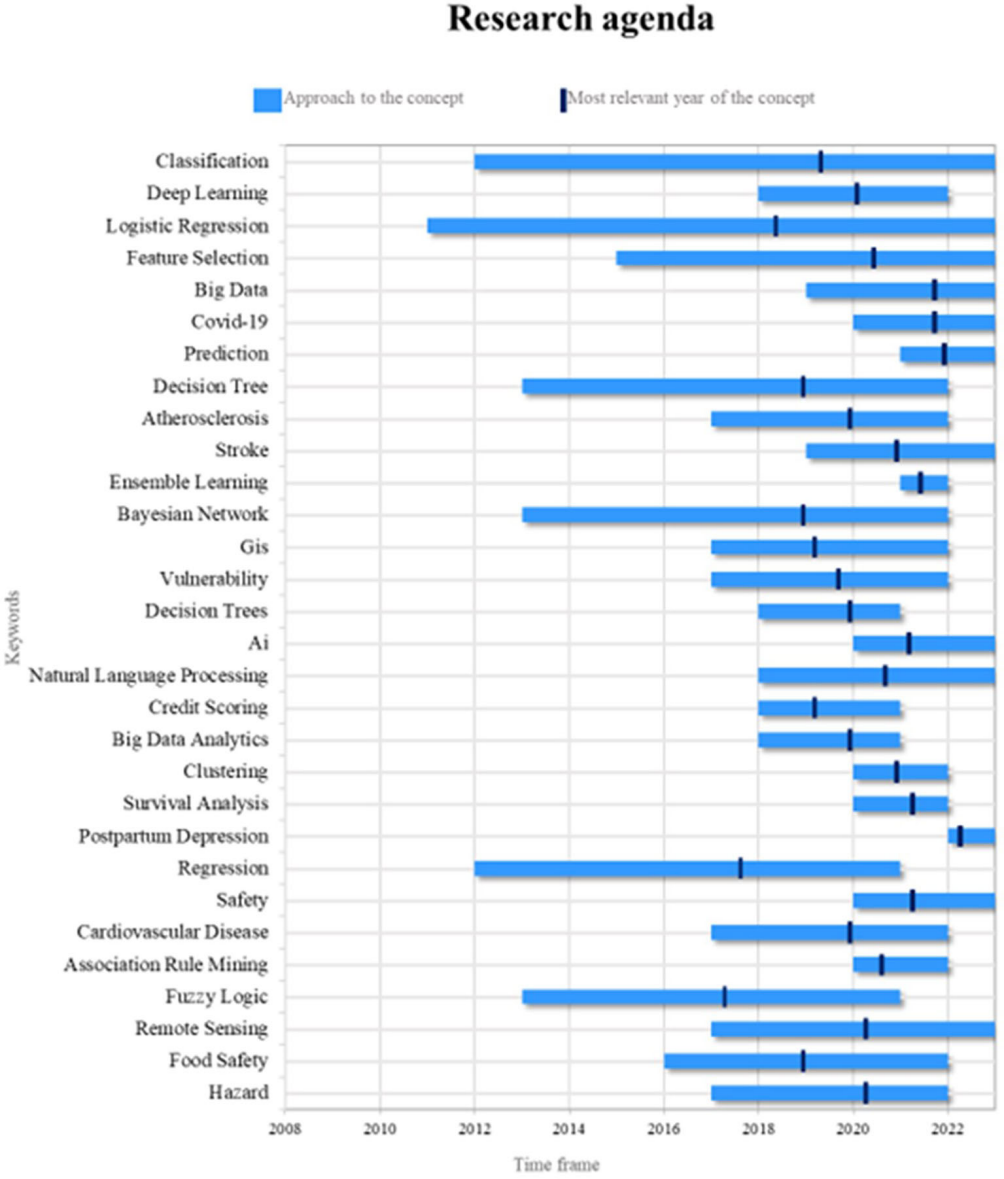
Agenda investigativa. Elaboración propia a partir de Scopus y Web of Science.

Lastly, Figure 9 outlines a suggested research roadmap for this bibliometric analysis, aiming to provide a reference for other scholars conducting future scientific investigations on stereotypes categorized as current, emerging, and trending. To achieve this, two key aspects are analyzed: (1) the period during which the term has appeared in the literature and (2) the year of highest significance regarding scientific output, the latter highlighting the timeframe when the term played a more prominent role in academic work and was also examined in the most recent year.

In this sense, it is noted that classification is a fundamental component in the application of machine learning to risk management, as it allows elements to be assigned to predefined categories based on their characteristics; currently, classification is widely used to identify patterns of risk behaviour and predict future outcomes in various fields, from credit analysis to fraud detection. For future research, more advanced classification approaches, such as deep learning, can be explored to improve the accuracy and efficiency of risk models, and it is important to investigate how classification can be integrated with other machine learning techniques, such as feature selection and logistic regression.

Logistic regression is another valuable tool in the application of machine learning to risk management today; it is used to predict the probability of an event occurring, which is useful for risk assessment and informed decision making. Future research can explore how logistic regression can be improved by incorporating more advanced techniques, such as regularization and hyperparameter optimization, to obtain more accurate and robust models. Similarly, it is important to explore how logistic regression can be applied in specific risk management contexts, such as early disease detection or financial loss prediction, and how it can be integrated with other machine learning techniques to improve its performance and usefulness.

Due to its ability to anticipate future events, prediction has become an emerging and relevant term in the field of study, and today prediction is applied in various fields, such as the estimation of climatic risks, the analysis of market trends, and the prediction of health crises. Future research can explore how the use of deep learning and big data techniques can improve the accuracy and predictability of models. It is also important to investigate how prediction can be applied to address emerging risks, such as those associated with the Covid-19 pandemic, and how predictive models can be developed that are adaptable to changing and variable environments.

The application of machine learning to the management of risks associated with Covid-19 has become highly relevant due to the need to address the challenges posed by the pandemic and is currently being used to predict the spread of the virus, identify at-risk groups and analyze epidemiological data. Future research can explore how machine learning can contribute to improving early detection of outbreaks, optimizing vaccination strategies and supporting public health decision making. It is also important to explore how the machine learning approaches used for Covid -19 can be applied to other infectious diseases, and how they can be adapted to address future health crises.

The use of big data has been driven by the increasing availability and accessibility of large amounts of data and is currently being applied to improve the accuracy and scalability of risk models, particularly in areas such as information security and risk management. of natural disasters. In future research, it is possible to see how big data can be integrated with advanced machine learning techniques, such as deep learning, to make the most of the available information, just as it is relevant to investigate how to address the challenges associated with data processing and analysis. large data sets, such as data privacy and data quality, to ensure reliable and effective risk management outcomes.

The term security has gained importance in the identification and prevention of threats, and is now used in intrusion detection, occupational risk analysis and safety in transport systems. Future research can explore how deep learning and neural networks can improve the ability to detect and adapt to new security threats, and how machine learning can address cybersecurity challenges such as developing defense systems and protecting confidential data.

## Conclusion

According to the bibliometric analysis of publication frequency and validity, the years 2023, 2022, 2021 and 2020 were the most significant in terms of interest in the use of machine learning in risk management. This indicates a growing interest and understanding of the importance of using machine learning to address risk management challenges in various sectors. It also suggests that there has been a significant increase in recent years in scientific production on this topic.

The scientific literature on the use of machine learning concludes that for risk management it has also shown cubic exponential growth, reflecting a constantly expanding field of study. This pattern of growth indicates that the topic has gained relevance and acceptance within the scientific community and will likely remain a busy and productive area of research for the foreseeable future.

The authors Laird, Suri and Saba, who have made significant contributions to the development of the literature, serve as the main research references in this area. The journals Science of the Total Environment and Computers and Industrial Engineering were also cited as important sources of literature on the subject. China, South Korea and the United States are the top three producers of scientific research, demonstrating their commitment to studying the use of machine learning for risk management.

The thematic development of the literature has changed significantly; we can conclude that in the past it focused on stacking, but now it focuses more on issues such as urban trees and Covid-19. This indicates the need to continue to explore new application areas and to modify risk management tactics to address current issues.

The analysis of thematic clusters has identified a consolidated set of terms with a strong conceptual affinity. Examples include random forest, machine learning algorithm, credit risk assessment, support vector machine, logistic regression and SVM. These fundamental findings provided a solid framework for further study and identified areas that should be prioritized for risk management modelling and methodologies.

In addition to the above findings, it is concluded that bibliometrics show that emerging keywords such as prediction, postpartum depression, Covid-19, big data, and security reflect highly relevant topics in the use of machine learning in risk management. These new ideas represent an expanding field of research and present opportunities to address specific problems, such as predicting future events, managing public health risks during pandemics, and incorporating big data to improve decision making.

Meanwhile, analysis of popular search terms such as prediction, big data, feature selection and deep learning highlights the importance of ongoing research in these areas to enhance and advance machine learning applications in industry. Risk management. These developed and extended ideas are trends in the development of methodologies and techniques applied to the difficulties of risk management in different context.

## Ethics and consent

Ethical approval and Consent were not required.

## Data Availability

No data are associated with this article. Zenodo: Machine Learning Applications in Risk Management: Trends and Research Agenda. https://doi.org/10.5281/zenodo.14841885. ^51^ The project contains the following extended data:
1. Dataset.xlsm (Raw data supporting the findings of this study). 2. PRISMA Checklist.docx (Checklist detailing compliance with PRISMA 2020 guidelines). 3. PRISMA flowchart.jpg (PRISMA flowchart) The data and materials are publicly available under a Creative Commons Attribution 4.0 International (CC BY 4.0) license. PRISMA Checklist for Machine Learning Applications in Risk Management: Trends and Research Agenda. https://doi.org/10.5281/zenodo.14841885. ^51^
