# Machine learning applications in risk management: Trends and research agenda

**DOI:** 10.12688/f1000research.161993.2

**Published:** 2025-04-07

**Authors:** Alejandro Valencia-Arias, Jesus Alberto Jimenez Garcia, Erica Agudelo-Ceballos, Aarón José Alberto Oré León, Ezequiel Martínez Rojas, Julio Leyrer Henríquez, Diana Marleny Ramírez-Ramírez

**Affiliations:** 1 Escuela de Ingeniería Industrial, Universidad Senor de Sipan, Chiclayo, 14001, Peru; 2 Dirección de Planificación y Desarrollo Institucional, Universidad Senor de Sipan, Chiclayo, 14001, Peru; 3 Departamento de Ciencias Administrativas, Instituto Tecnologico Metropolitano, Medellín, 50010, Colombia; 4 Instituto de Investigación de Estudios de la Mujer, Universidad Ricardo Palma, Santiago de Surco, 15039, Peru; 5 Vicerrectoría de Investigación e Innovación, Universidad Arturo Prat, Iquique, Tarapacá Region, Chile; 6 Universidad Ricardo Palma, Lima, Peru; 7 Ciencias económicas y administrativas, Instituto Tecnologico Metropolitano, Medellín, 50010, Colombia

**Keywords:** Decision Making, Random Forest, Big Data, Deep Learning, Security

## Abstract

Risk management has become a foundational aspect in numerous industries, propelling the implementation of machine learning technologies for impact assessment, prevention, and decision-making processes. Nevertheless, lacunae in the extant literature persist, particularly with regard to the identification of emergent trends and transversal applications. This study addresses this limitation through a bibliometric analysis of scientific production in Scopus and Web of Science, adhering to the PRISMA-2020 declaration. The findings reveal a substantial growth in publications on machine learning applied to risk management, with an increase of 98.99% between 2018 and 2023. China, South Korea, and the United States are identified as the primary research-producing countries. The analysis also identifies emerging trends, such as the application of machine learning in the evaluation of urban trees and the management of risks associated with the pandemic of severe acute respiratory syndrome (SARS-CoV-2). Key terms include random forest, support vector machines (SVM), and credit risk assessment, while terms such as prediction, postpartum depression, big data, and security emerge as new areas of study. Furthermore, there is a transition from traditional approaches such as stacking to advanced deep learning and feature selection techniques, reflecting the evolution of the discipline.

## Introduction

Risk management is a very important activity for various industries and economic sectors that seek to detect, evaluate, and reduce uncertainties that may have a negative impact on the achievement of organizational objectives. Over the years, the exponential growth of data and the complexity of risks have challenged traditional risk management methodologies, thus the adoption of automatic learning or machine learning techniques has been considered an excellent tool. to address challenges in risk management. ^1, 2^

Conversely, machine learning represents a crucial component of artificial intelligence, facilitating the analysis of copious quantities of data, the identification of patterns, and the discovery of hitherto unrecognized insights. This markedly enhances the capacity to prevent and make decisions in risk management. The application of machine learning in risk management within supply chains has been well documented. For example, ^3^ utilised a deep-learning-based dual-stage PLS-SEM-ANN analysis to improve supply chain agility and resilience. A novel risk assessment method driven by big data focused on supply chains in the area of economic promotion at airports has demonstrated how machine learning contributes to the efficiency and safety of the transportation and distribution of goods. ^1^ Moreover, machine learning’s capacity for prediction in the context of supply chain risk management is of paramount importance in enabling the proactive identification of potential disruptions and the subsequent adjustment of logistics strategies. This underscores the value of machine learning in enhancing operational continuity and decision-making processes. ^4^

Furthermore, machine learning has also become a significant tool in the field of medical risk management, offering the potential to enhance the safety and quality of healthcare. For example, the application of machine learning in the field of diabetic healthcare has enabled the development of more precise predictive models, thereby facilitating more informed clinical decision-making and the creation of personalized treatment plans. ^5^ Moreover, sophisticated risk assessment instruments that employ machine learning integrate an array of predictive models to furnish precise and adaptable assessments of perioperative risks associated with medical procedures, thus facilitating the formulation of bespoke interventions for patient safety. ^6^ Furthermore, machine learning has been employed to forecast the likelihood of inpatient falls by examining both intrinsic and extrinsic variables, thereby underscoring its far-reaching influence on the domain of healthcare risk management. ^7^

Notably, machine learning is also having a significant impact on risk management in the financial sector. The use of machine learning for risk assessment and management in financial portfolios with high-dimensional problems has provided a deeper and more efficient understanding of the risks associated with investments and portfolios. ^8^ Similarly, machine learning has proven to be a valuable tool in credit risk assessment, as it can leverage human experience and computational intelligence to improve predictive accuracy by combining expert knowledge with genetic algorithms in credit selection characteristics for credit risk assessment. ^9^

The application of machine learning to risk management has gained significant importance in various fields such as medicine and engineering. Advances in this area have allowed the development of more accurate and effective risk assessment tools, which implies more informed decision making with less margin of error; examples include the integration of machine learning-based predictions for perioperative risk management ^6^ and the identification of perineural invasion in head and neck squamous cell carcinoma. ^10^

Machine learning has emerged as a novel approach to risk management, a field that has historically relied on predefined statistical models and human judgment. In contrast to these traditional methods, machine learning facilitates more accurate and expeditious risk detection by processing vast volumes of data in real time. This advantage is exemplified in the financial sector, where conventional credit assessment models, predicated on fixed rules and credit scores, have been eclipsed by machine learning algorithms that analyze intricate patterns of financial behavior. This has led to a substantial reduction in the false positive rate in fraud detection. ^11^ In the domain of healthcare, conventional methods for predicting cardiovascular diseases have been complemented, and in some cases, surpassed by machine learning (ML) models that integrate clinical and genetic data, thereby enhancing diagnostic accuracy and personalizing treatments. ^4^ In a similar vein, within the manufacturing sector, the efficacy of AI-driven predictive maintenance systems has been demonstrated to exceed that of conventional preventive maintenance methodologies. These systems are capable of anticipating failures based on operational data, as opposed to the more rigid, schedule-based approaches that have historically prevailed. ^12^ These examples underscore the potential of artificial intelligence to not only optimize risk management but also to foster a more proactive and adaptive approach, thereby minimizing costs and enhancing decision-making processes across diverse sectors.

The interpretation of the models is an essential aspect in areas where decisions can have guidelines for human life because the ability to understand and justify the results of the model is essential to gain the confidence of the professionals and patients involved. In the decision-making process, it is therefore novel to incorporate the interpretability of models for cardiovascular risk assessment using machine learning, which allows a better understanding of how inflammation biomarkers influence risk estimation. ^2^

Furthermore, in non-medical areas such as accident risk management, research on the application of machine learning has made significant progress in recent years, as the application of these techniques in accident prevention and mitigation can have a significant impact on industrial safety and the protection of people and the environment. However, there are still important gaps that justify conducting an exhaustive bibliometrics, one of the main gaps being the adaptation of machine learning models to specific contexts. ^13^

However, In Malaysia, an accident risk analysis study was conducted, which highlights the importance of continuing to study and address the challenges that still exist in the implementation of these technologies, while ensuring that they are ethical, fair models and based on high quality data in order to achieve a positive and significant impact on society, particular challenges were identified in the application of supervised techniques, where the need to develop approaches and models that take into account the cultural, regulatory and environmental characteristics of each context stands out, allowing for more effective and personalized risk management. ^13^

A significant research gap in the domain of risk management pertains to the early detection of emerging risks across various sectors. Conventional risk management techniques frequently depend on historical data and predefined models, thereby constraining their capacity to anticipate unanticipated threats. In the field of healthcare, for instance, machine learning has demonstrated efficacy in predicting help-seeking behavior among women with postpartum depression, underscoring its potential to identify emergent risks before they escalate into critical situations. However, significant limitations persist in the application of these techniques to other fields, including industrial safety, environmental risk, and finance. These limitations include data scarcity, contextual adaptability, and the challenge of integrating domain-specific knowledge into predictive models.

Addressing these issues requires further research to enhance the applicability and reliability of machine learning in dynamic and complex risk environments. Despite advancements in the application of machine learning for risk management, several critical challenges hinder its widespread adoption. A significant challenge pertains to the interpretability of models, as black-box algorithms often lack transparency, hindering the trust decision-makers in highly regulated sectors such as healthcare and finance can place in model predictions. Additionally, biases embedded in training data can result in unfair or inaccurate risk assessments, thereby exacerbating inequalities in risk mitigation efforts. Regulatory concerns also pose significant barriers, as the absence of standardized guidelines for algorithmic decision-making complicates compliance and limits implementation. To address these challenges, this study aims to identify emerging research trends, key opportunities, and innovative methodologies that can enhance the integration of machine learning into risk management while ensuring ethical, transparent, and effective applications.

While existing machine learning models and traditional risk management strategies have demonstrated potential in improving decision-making and risk mitigation, a more comprehensive investigation is required to optimize their integration. The effective combination of these approaches remains an open question, particularly regarding how to balance machine learning’s predictive power with the contextual knowledge embedded in conventional risk management frameworks. This study, therefore, seeks to examine research trends in machine learning for risk management from 2007 to 2023, providing a structured research agenda that outlines key areas for future exploration. To achieve this, the study addresses the following research questions:

- What are the years when there has been more interest in machine learning in risk management?
- What is the growth rate of scientific articles on machine learning in risk management?
- What are the main research references on machine learning in risk management?
- What is the thematic evolution derived from the scientific production on machine learning in risk management?
- What are the main thematic clusters on machine learning in risk management?
- What are the growing and emerging keywords in the research field of machine learning in risk management?
- Which topics are positioned as protagonists for the design of a research agenda on machine learning in risk management?

This article is structured in such a way that it begins with a review of the literature considered relevant in the field of the use of machine learning in risk management. Subsequently, the methodological section details the study design and the procedures used to collect and analyze the data in order to answer the questions raised above. The results are presented in the results section, where relevant information and statistics are presented, and their implications are discussed in the final section of the article.

## Methods

To achieve the objective of this research, an exploratory methodology is proposed, based on secondary research sources, through the performance of a bibliometric analysis that allows an evaluation of the scientific literature, specifically in relation to the use of machine learning in risk management. For this purpose, the parameters, or protocols of the international declaration PRISMA-2020 ^14^ will be followed. The selection of a bibliometric methodology and the implementation of the PRISMA 2020 framework are responses to the necessity of a systematic and objective evaluation of scientific output related to machine learning in the context of risk management. In contrast to other methodologies, such as systematic mapping studies, bibliometrics facilitates the identification of patterns within the literature, the discernment of emergent trends, and the analysis of the relationships between authors and publications through the utilization of quantitative indicators. The PRISMA 2020 framework was selected due to its rigorous structure for collecting and filtering studies, ensuring transparency and reproducibility of the analysis.

### Inclusion criteria

The inclusion criteria for this bibliometric study on machine learning in risk management focus on two main aspects in document titles and metadata. First, records must contain terms related to “risk management” and its synonyms, such as “risk control.” Second, documents combining “risk management” with “risk assessment” and “risk analysis” are included, as these concepts provide a comprehensive view of the subject.

The exclusion process involves three phases. In the first phase, records with indexing errors are discarded. The second phase eliminates documents without full text access, applicable mainly to systematic literature reviews, since bibliometric analyses rely on available metadata. Finally, the third phase removes conference proceedings and documents with incomplete indexing that are not relevant, ensuring the quality and relevance of selected documents. These rigorous criteria aim to yield reliable results regarding the application of machine learning in risk management. ^14^

The inclusion and exclusion criteria were meticulously designed to ensure the robustness and relevance of the literature analyzed. The incorporation of key terms such as “risk management,” “risk assessment,” and “risk analysis” ensures comprehensive coverage of studies addressing risk management from diverse perspectives. The exclusion of documents with restricted full-text access responds to the necessity for complete metadata for accurate bibliometric analysis. Furthermore, the elimination of conferences and documents with incomplete indexing aims to reduce bias and enhance the quality of the data analyzed.

### Sources of information

In conducting this bibliometric study on machine learning in risk management, the Scopus and Web of Science databases were selected as the primary sources of information. ^15^ These databases provide comprehensive coverage of scientific journals, conferences, and patents, ensuring accurate and reliable indexing of articles. The combination of these databases will yield a more comprehensive and representative overview of the scientific output on this topic.

### Search strategy

In order to carry out the bibliometric search on the application of machine learning in risk management in the Scopus and Web of Science databases, two specialized search equations were designed, corresponding to the defined inclusion criteria and the search characteristics of each database.

For the Scopus database, the search formula was structured as follows TITLE (“risk management” OR “risk assessment” OR “risk analysis”) AND TITLE (“machine learning” OR “artificial intelligence” OR “predictive modelling” OR “data mining”). This formula combines the terms related to risk management in the title of the documents using the OR operator and similarly groups the terms related to machine learning. This combination of terms in the title of the documents makes it possible to obtain an exhaustive and precise search on the application of machine learning to risk management in the Scopus database.

On the other hand, for the Web of Science database, the search equation was formulated in a similar way: TI=(“risk management” OR “risk assessment” OR “risk analysis”) AND TI=(“machine learning” OR “artificial intelligence” OR “predictive modelling” OR “data mining”). In this equation, synonyms related to risk management and terms related to machine learning are again grouped using the OR operator in the title of the documents. This search structure makes it possible to obtain relevant and up-to-date results in the Web of Science database.

The utilization of specific search equations is pivotal in this context, as they serve to amalgamate pivotal terminology associated with risk management and machine learning within the document title. The employment of the OR operator facilitates the aggregation of synonyms within each category, while the AND operator ensures the interrelation of these terms. This methodological approach guarantees an exhaustive and precise search, with a specific focus on studies that address the application of machine learning in the domain of risk management.

The search equations were meticulously crafted to optimize the precision and scope of the relevant scientific literature, thereby ensuring that the bibliometric analysis comprehensively mirrors the current state of knowledge concerning the application of machine learning in risk management.

### Data management

During the development of the bibliometric analysis on the use of machine learning in risk management, Microsoft Excel was used to extract, store and process information from various databases. In addition, both the free software VOSviewer and Microsoft Excel were used to visualize the bibliometric indicators obtained. ^16^ The combination of both tools made it possible to create representative and accurate graphs of the collected data, which facilitated the analysis and presentation of the research results.

### Selection process

The PRISMA 2020 statement underscores the necessity of employing an automated classifier in the study selection process and validating its performance to mitigate the risks of missed or incorrectly classified studies. ^14^ In this study on machine learning in risk management, researchers employed Microsoft Excel automation tools developed in-house to apply inclusion and exclusion criteria independently. This approach was designed to reduce the likelihood of overlooking pertinent studies or misclassification by aligning the results of different reviewers.

### Data collection process

This study used Microsoft Excel as an automated tool to facilitate the data collection and organization process. All study authors acted as reviewers and independently validated the data. In addition, a collective data confirmation process was carried out to ensure sufficient verification until absolute convergence of the results obtained was achieved.

A thorough evaluation of potential biases in the study selection process has been conducted, encompassing considerations of selection bias, publication bias, and the potential exclusion of pertinent studies. To address these biases, automated tools were integrated into Microsoft Excel to systematically implement inclusion and exclusion criteria. Moreover, the reliability of the automatic classifiers was validated by comparing their outcomes with a manual review conducted by multiple authors, ensuring the uniformity in the selection of documents.

### Data elements

In this bibliometric study on machine learning in risk management, comprehensive data searches were conducted to identify and collect all relevant articles. A specialized search application was developed for each database to ensure comprehensive inclusion. However, to ensure the coherence and relevance of the study, any missing or unclear information was excluded, as were texts deemed to be of no relevance. This approach ensured that the research was focused on pertinent data, thereby aligning it with the study’s stated purpose and scope.

### Assessment of the risk of bias in the studies

A substantial emphasis was placed on the evaluation of the potential for bias in the selected studies. All authors contributed to this assessment through the utilisation of an enhanced automated Microsoft Excel tool, thereby ensuring consistency and accuracy. This collaborative approach, coupled with a reliable tool, was employed with the objective of minimising the potential for bias, thereby enhancing the reliability and validity of the research outcomes.

### Impact measures

This bibliometric study on machine learning in risk management acknowledges the paucity of analysis of diverse impact measures, which are more frequently employed in primary research. In accordance with the nature of secondary research, the study employed measures such as the number of publications and citations to assess relevance and impact. Furthermore, the study employed a temporal analysis of keyword usage to identify emerging trends. The manipulation and analysis of the data were conducted using Microsoft Excel, while VOSviewer ( VOSviewer - Visualizing scientific landscapes) was employed to ascertain the thematic associations between the documents. This methodological approach afforded a comprehensive understanding of scientific production in this field, thereby enhancing the research findings based on secondary sources.

### Synthesis methods

The bibliometric indicators of quantity, quality, and structure, as outlined by, ^17^ were automatically applied using Microsoft Excel, thereby streamlining the analysis process. The automation facilitated efficient information processing and ensured consistency in the application of the indicators, thereby enhancing the quality and reliability of the bibliometric research on machine learning in risk management.

### Assessment of reporting bias

It is of paramount importance to assess the risk of bias, given the potential for gaps in the synthesis of results. Bias may result from the use of specific synonyms in thesauri, such as IEEE, which can influence the criteria for inclusion, search strategies, and data collection. Furthermore, the use of conference proceedings may result in the omission of pertinent information due to incomplete indexing and the exclusion of irrelevant materials. It is imperative to consider these factors in order to achieve a more accurate and comprehensive evaluation of the collected data.

### Evaluation of certainty

**Figure 1.**
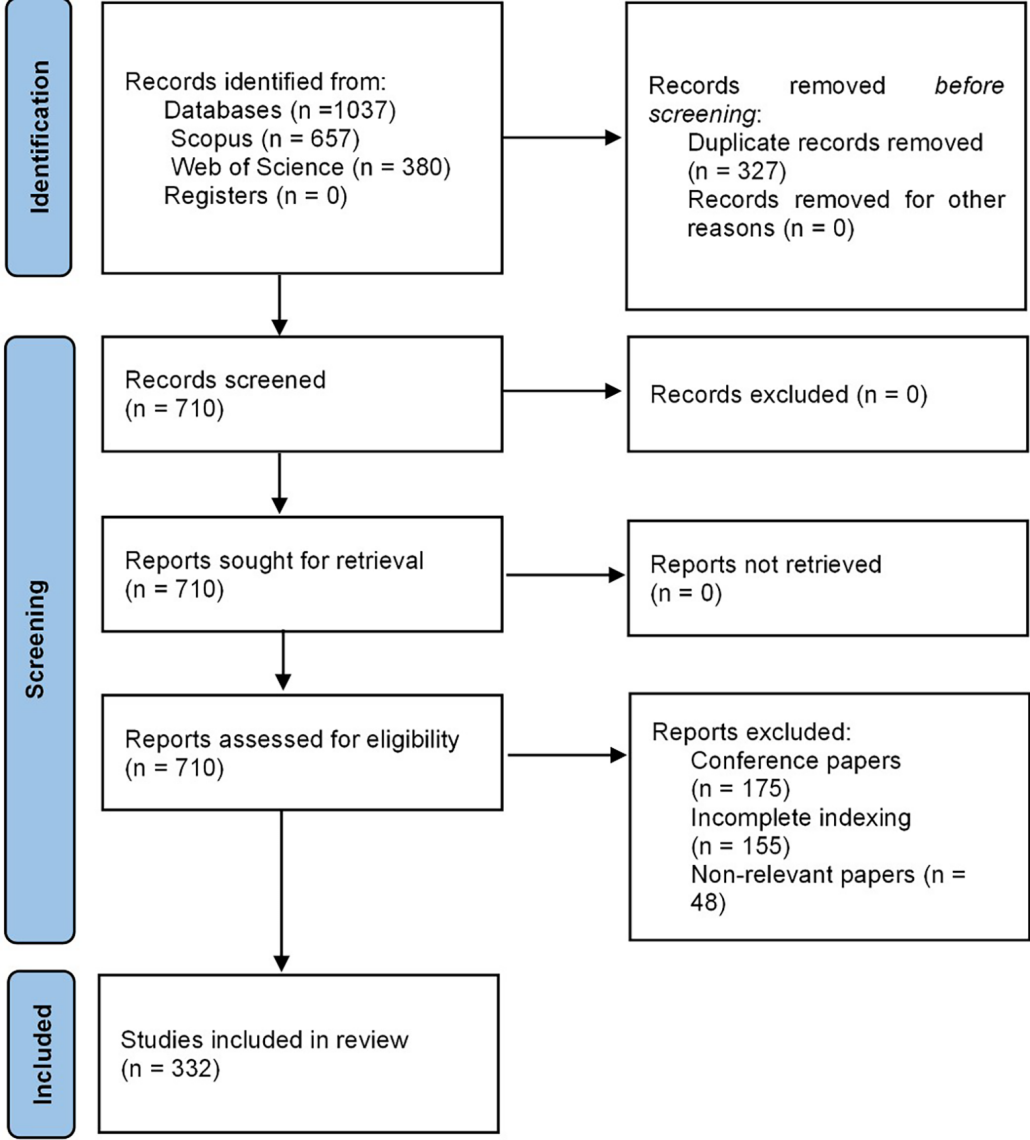
PRISMA flow chart. Own elaboration from Scopus and Web of Science.

Is comprehensive in its assessment of the certainty of the evidence. It considers a number of factors, including the independent application of inclusion and exclusion criteria, bibliometric indicators, and potential methodological biases. The discussion section addresses the limitations of the studies included in the review, thereby enhancing transparency. This comprehensive methodology is designed to yield a robust and reliable evaluation of the evidence on the topic. In the following Figure 1, the entire methodological design is evident from the PRISMA-2020 flow chart.

## Results

**Figure 2.**
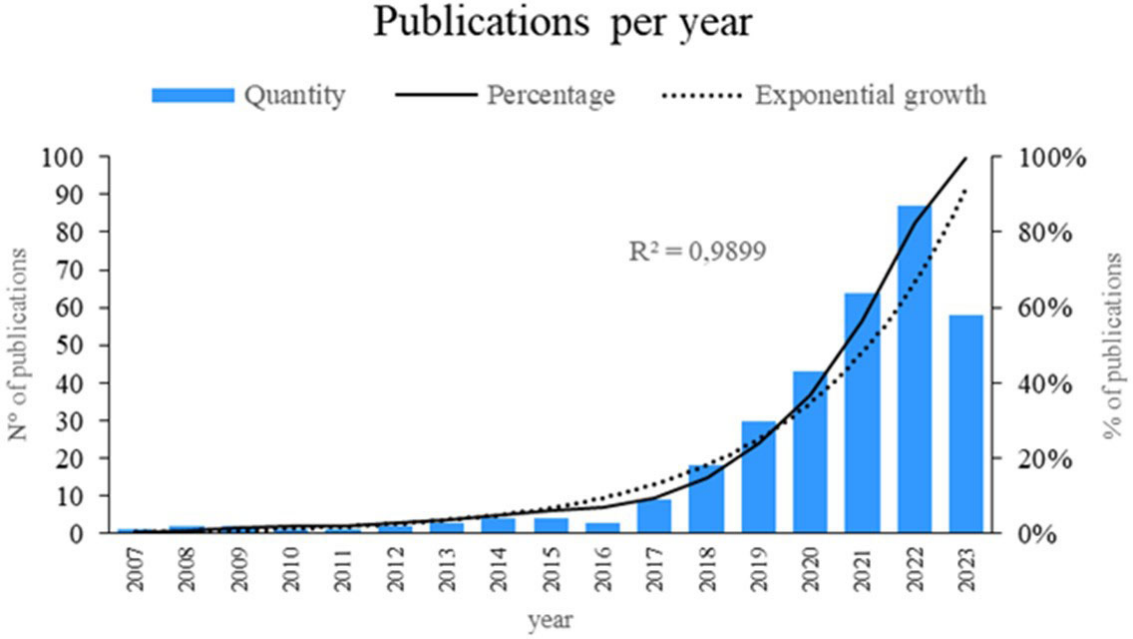
Publications by year. Own elaboration from Scopus and Web of Science.

Figure 2 presents a comprehensive analysis of the scientific literature in the field. The results indicated an exponential growth of 98.99% in published articles, representing a significant increase over time. The years 2023, 2022, 2021, and 2020 were the most notable in terms of publication output, reflecting a growing interest in the topic. These findings provide a clear and up-to-date perspective on the current state of the scientific landscape regarding the application of machine learning in risk management, thus contributing to the advancement of knowledge in this area.

**Figure 3.**
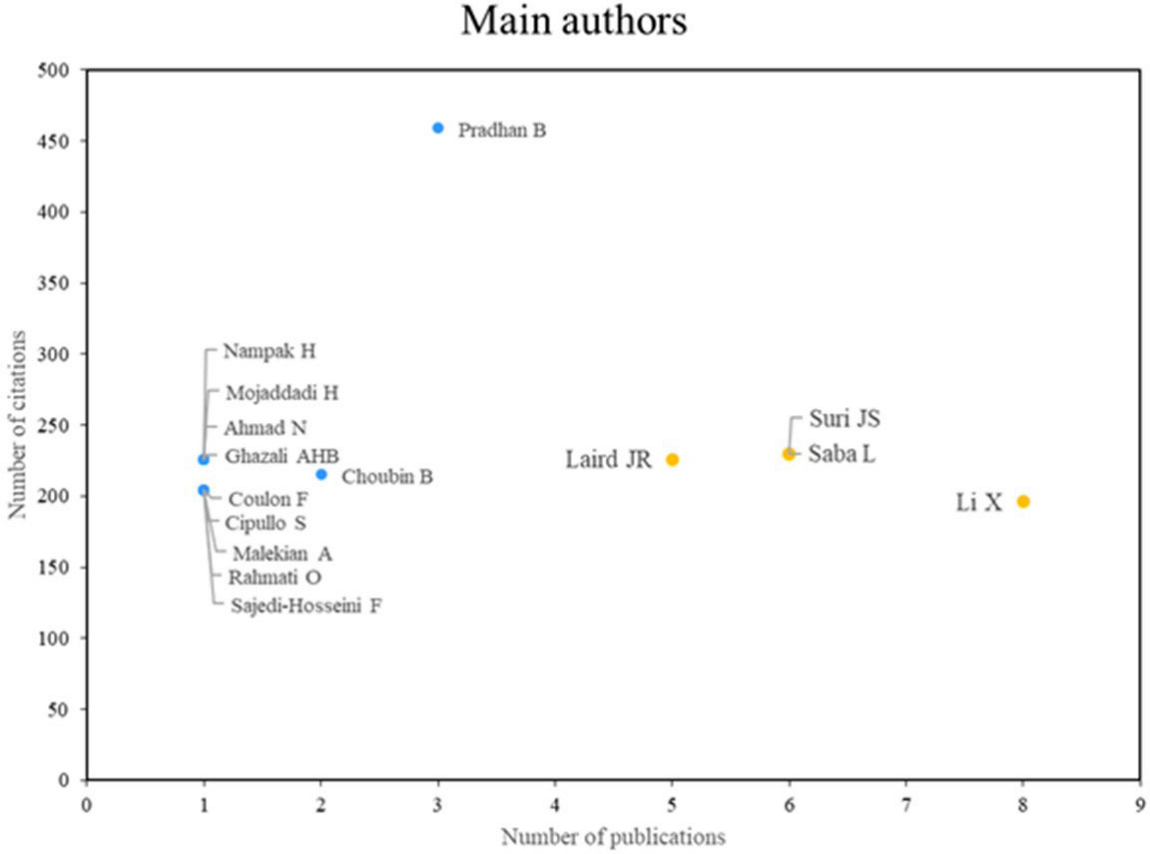
Main authors. Own elaboration from Scopus and Web of Science.

Figure 3 illustrates the two principal categories of distinguished authors. The first group, comprising Laird, Suri, Saba, and Li, is distinguished by high scientific productivity and a notable research impact, as evidenced by a substantial body of relevant publications. The second group, comprising researchers such as Pradhan and Choubin, is distinguished by their impactful contributions, despite exhibiting lower scientific productivity. Both groups demonstrate multifaceted contributions to the field.

**Figure 4.**
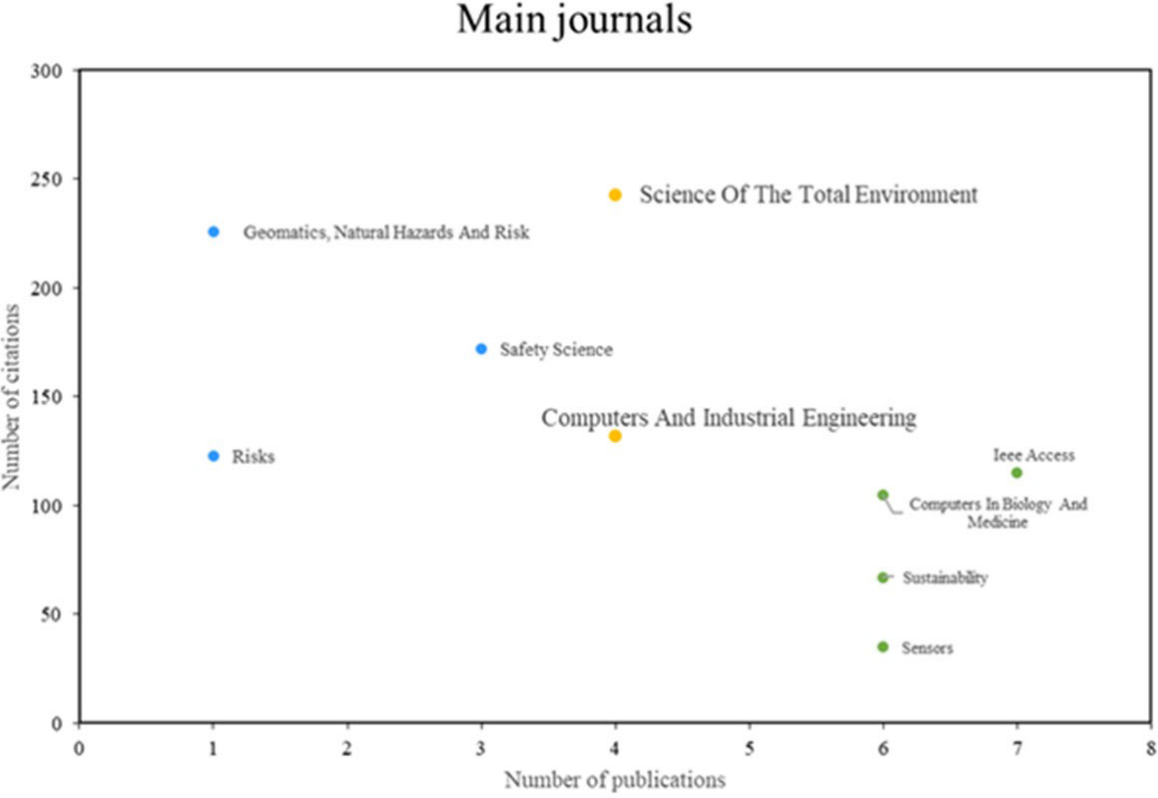
Main journals. Own elaboration from Scopus and Web of Science.

As illustrated in Figure 4, three principal categories of noteworthy journals were identified. The initial group comprises journals such as Science of the Total Environment and Computers and Industrial Engineering, which demonstrate remarkable productivity and impact, publishing a substantial number of pertinent articles and receiving a considerable number of citations. The second group includes journals such as Geomatics, Natural Hazards, and Risk and Safety Science, which are renowned for their high impact despite exhibiting lower productivity. Lastly, the third group comprises journals such as IEEE Access and Sensors, which are distinguished by high scientific productivity, although they may exhibit a lower number of citations compared to other journals.

**Figure 5.**
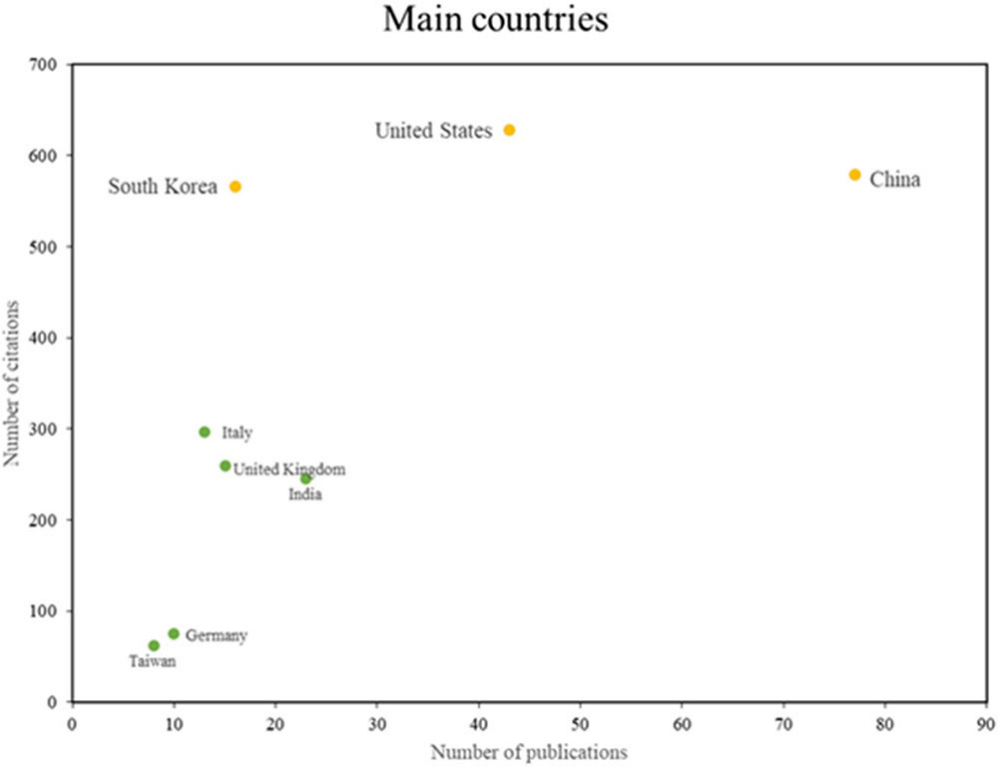
Main countries. Own elaboration from Scopus and Web of Science.

Figure 5 identifies two principal categories of countries that demonstrate particular excellence in this field. The initial group, which encompasses South Korea, the United States, and China, is distinguished by elevated levels of scientific productivity and impact, as evidenced by substantial research output and a multitude of citations. The second group, which includes countries such as Italy and India, exhibits robust scientific productivity but has not yet attained a comparable level of citations. This illustrates the diversity and global scope of research in this field, with different countries contributing distinctive strengths in productivity and impact.

**Figure 6.**
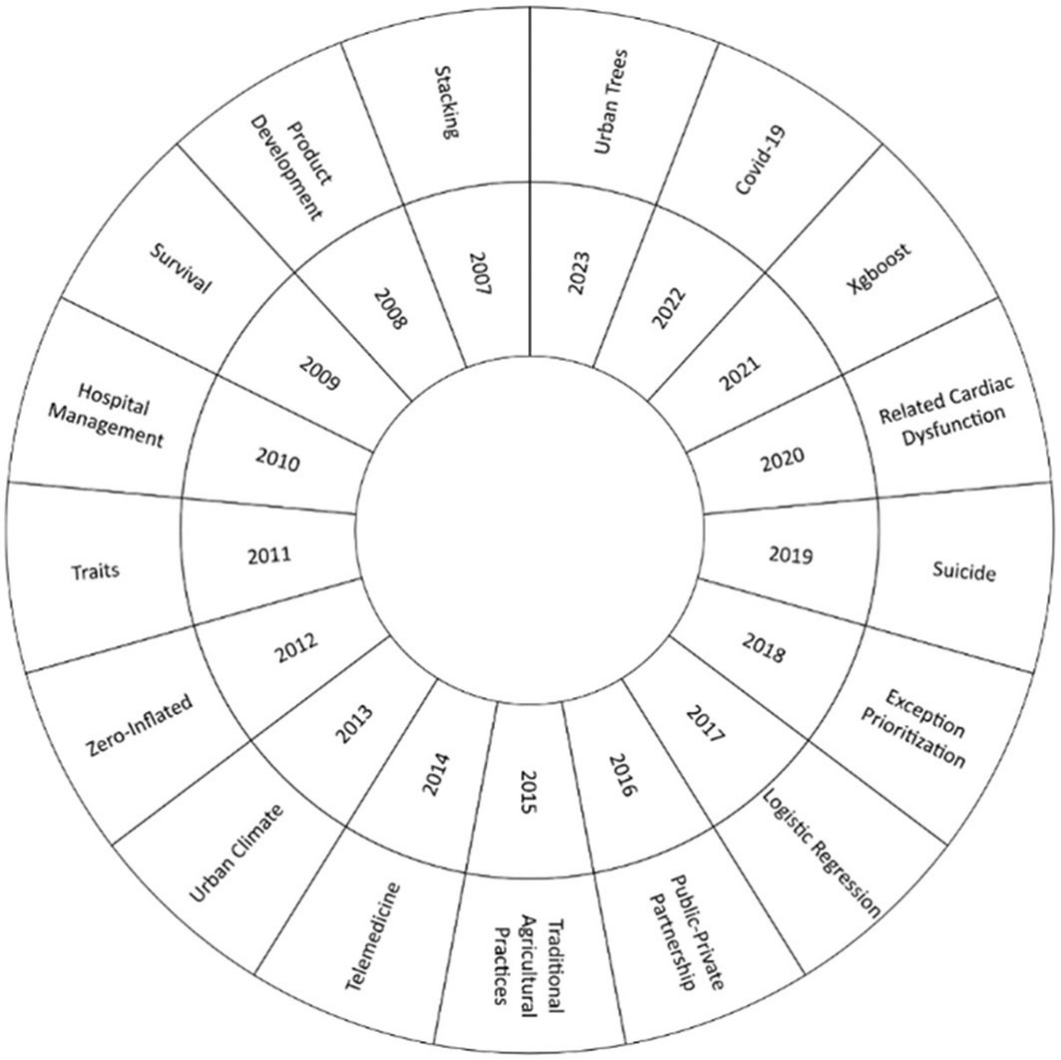
Thematic evolution. Own elaboration from Scopus and Web of Science.

In this bibliographic research, we examined how the machine learning approach to risk management has evolved over the years 2007 to 2023, as shown in Figure 6. The most frequent keyword in each year was analyzed to identify changes and trends in the field. At the beginning of the analysis, in 2007, the emergence of the term ‘stacking’ stood out. Over time, a significant thematic evolution was observed, with the emergence of relevant topics today, such as “Urban Trees”, “Covid-19”, “Xgboost”, “Related Cardiac Dysfunction” and “Suicide”.

**Figure 7.**
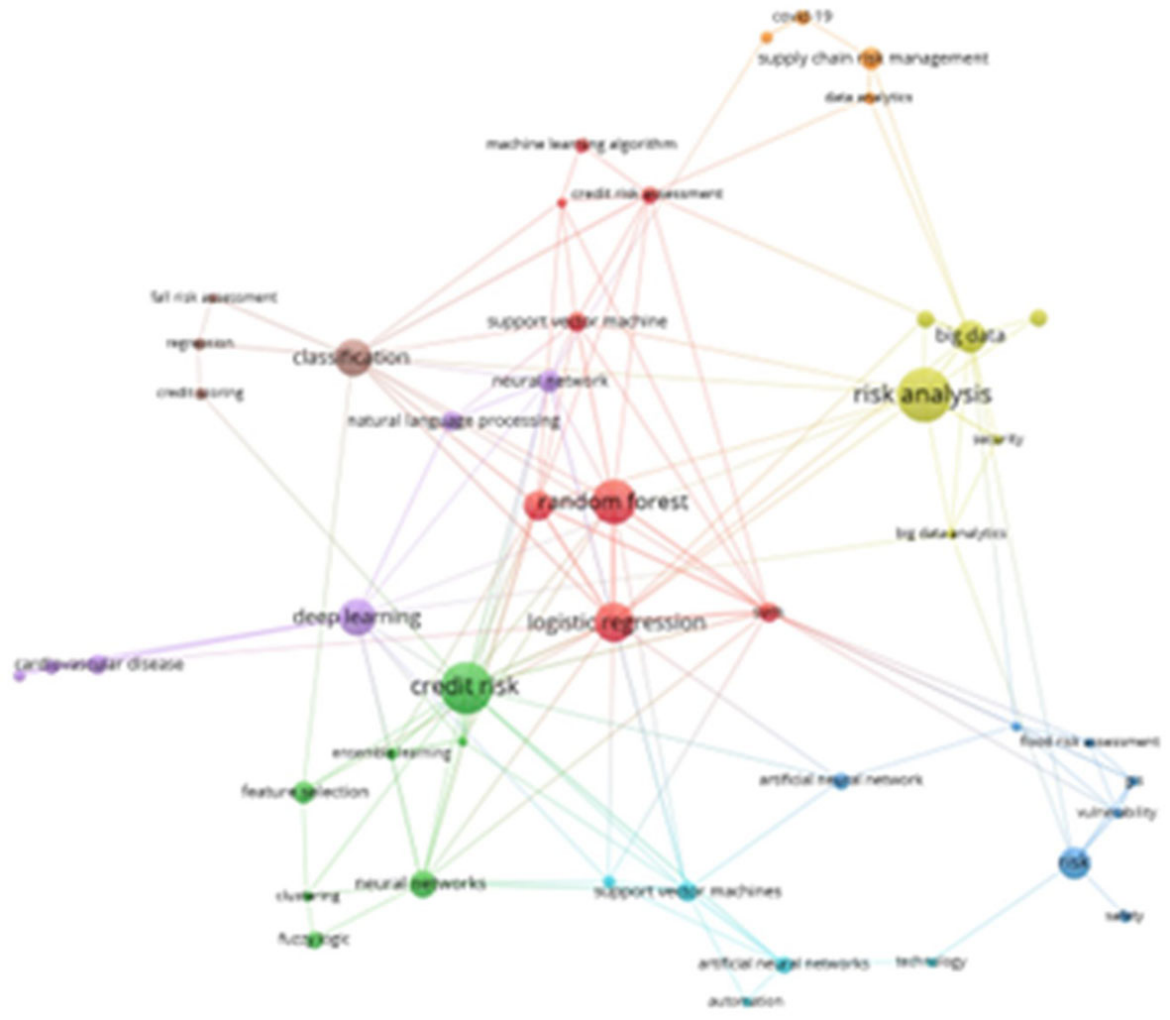
Keyword co-occurrence network. Own elaboration from Scopus and Web of Science.

The following presents an overview of a network of related keywords, which have been organized into eight thematic groups, as illustrated in Figure 7. The most prominent cluster, indicated in red, encompasses terms such as “Random Forest,” “Machine Learning Algorithm,” “Credit Risk Assessment,” “Support Vector Machine,” “Logistic Regression,” and “SVM.” The dark green cluster features terms such as “credit risk,” “ensemble learning,” “feature selection,” “neural networks,” “clustering,” and “fuzzy logic.” Additional clusters in lemon green, dark blue, light blue, purple, orange, and brown reflect various aspects of conceptual affinity within the field.

**Figure 8.**
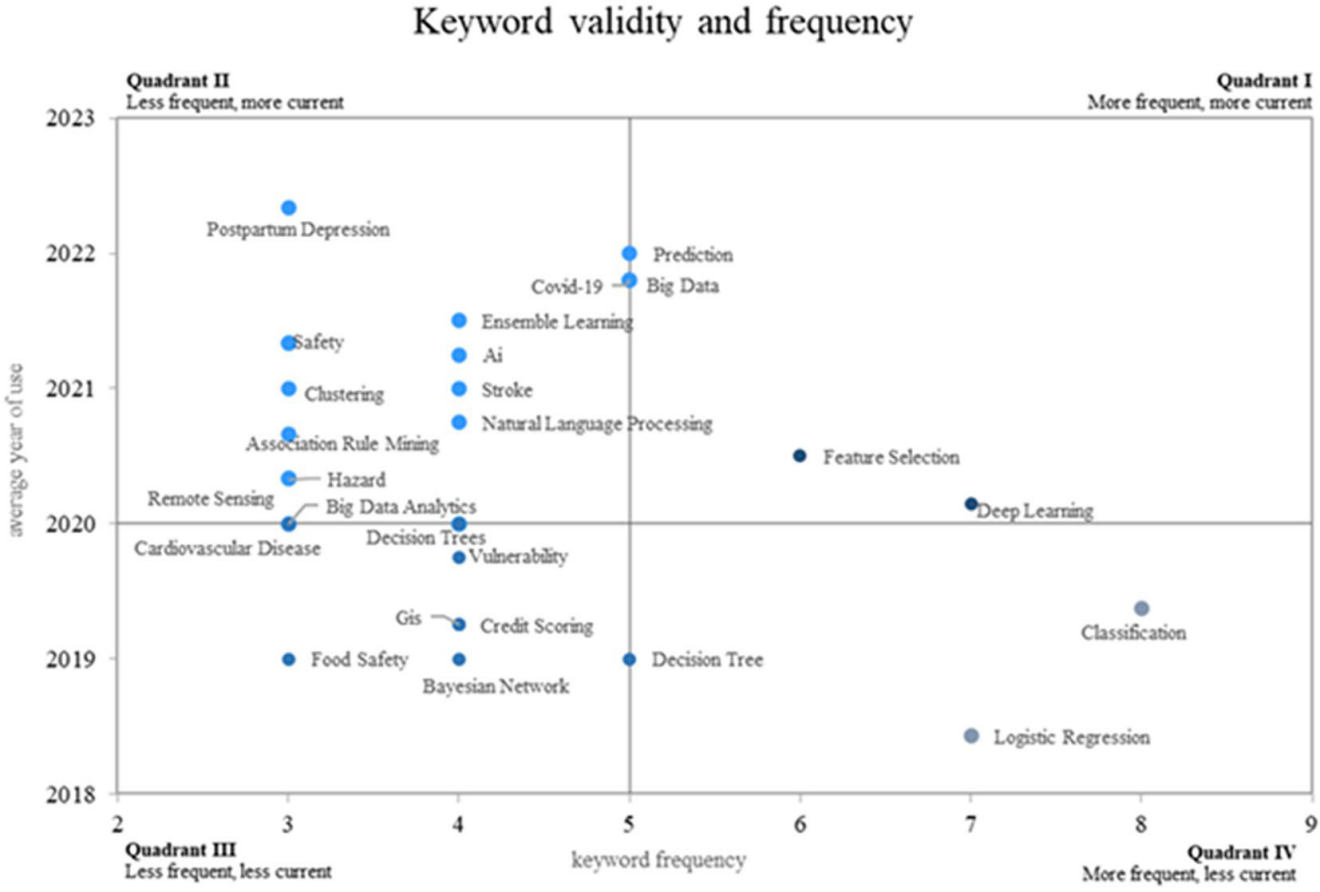
Validity and frequency of the keywords. Own elaboration from Scopus and Web of Science.

This research on the application of machine learning to risk management proposes a novel approach using a Cartesian plane that measures the frequency of use of keywords on the X-axis and the validity of use on the Y-axis, thus showing four different quadrants. as shown in Figure 8. Quadrant 4 contains descending concepts, including keywords such as classification, logistic regression, and decision tree. Quadrant 2 contains rare but highly topical words that are considered to be emerging, such as prediction, postpartum depression, Covid-19, big data and security. On the other hand, consolidated and growing terms such as prediction, big data, feature selection and deep learning are positioned in quadrant 1.

## Discussion

The discussion of the bibliometric analysis results presents an overview of the most salient findings, including annual scientific production, notable research references, the evolution of the subject, thematic clusters, and the frequency and validity of keywords. Furthermore, the section classifies fundamental keywords based on their function, examines the practical implications of such classification, discusses the limitations of the approach, and identifies research gaps. In conclusion, the paper puts forward a primary research agenda for the future, with a particular focus on machine learning in risk management.

### Analysis of the growth of scientific literature on the application of machine learning in risk management

A review of scientific output revealed a notable surge in publications pertaining to the deployment of machine learning in risk management between 2020 and 2023 ( Figure 2). For example, in 2020, a data mining-based framework for supply chain risk management was introduced. ^18^ Furthermore, an artificial intelligence approach for assessing the risk of infection with the novel coronavirus (2019-nCoV) in virtual medical visits was demonstrated. ^19^ In 2021, some authors investigated the potential of explanatory machine learning in credit risk management, ^20^ while others explored the use of hybrid artificial intelligence models for flood risk assessment in Quang Nam province, Vietnam. ^21^ These studies illustrate the accelerated evolution of machine learning applications across diverse domains, including supply chain management and public health.

In a continuation of this trend, a microbiological prediction model was developed for food risk analysis in 2022. This model is based on the Wiener process integrated with single-step kinetics. ^22^ Furthermore, deep learning was employed in the domain of supply chain risk management with the objective of enhancing agility, utilizing a dual PLS-SEM-ANN analysis. ^23^ By 2023, researchers had conducted a comprehensive study on the opportunities and challenges of supervised learning in maritime risk analysis. ^24^ Moreover, ^25^ conducted a literature review that underscored the significance of machine learning technology in bolstering supply chain risk management practices, proposing prospective avenues for future research to further integrate these sophisticated methodologies. The papers cited represent a sampling of the expanding body of research on the implementation of machine learning in risk management, addressing disparate topics and presenting innovative approaches that enhance the body of knowledge in this interdisciplinary field. Additionally, ^12^ conducted a comprehensive review of machine learning methods that have been specifically designed for engineering risk assessment. Their work illustrates the diverse applications and significance of machine learning in enhancing risk management frameworks across various industries.

### Analysis of research references on the application of machine learning in risk management

As for the main authors who stand out in terms of productivity and scientific impact in the application of machine learning in risk management, Laird, Suri and Saba, Li are shown in Figure 3. Laird, for his part, has excelled in research related to cardiovascular and cerebrovascular risk assessment using machine learning techniques; In a 2021 study, a multiclass machine learning approach to risk assessment of stroke and cardiovascular disease was presented, using predictors of carotid plaques with coronary angiography construct as the gold standard. ^26^ Also, Suri and Saba have contributed significantly in this field, as evidenced in. ^27^

For his part, author Li is considered a research benchmark in machine learning applications for risk assessment in process operations. In one of his studies, he presented a machine learning methodology for probabilistic risk assessment in gas leak incidents in underwater pipelines. ^28^ In addition, Li has worked on global flood risk assessment using machine learning models, applying several machine learning models to assess flood risk in global river basins. ^29^

On the other hand, Pradhan and Choubin stand out for their academic impact in research on risk assessment in natural disasters and water pollution, respectively. In a collaborative investigation, machine learning approaches were used to assess earthquake risk in Palu, Indonesia ^30^; similarly, they proposed a machine learning-based approach to assess the risk of nitrate contamination in groundwater ^31^; they also demonstrated the application of machine learning in urban flood risk assessment, integrating decision making and machine learning techniques. ^32^

Next, looking at the most prominent journals included in Figure 4, in terms of productivity and scientific impact, Science of The Total Environment and Computers & Industrial Engineering were found. The journal Science of The Total Environment has contributed significantly to the knowledge on this topic through research using machine learning approaches for earthquake risk assessment in Palu, Indonesia ^30^; machine learning has also been used for risk assessment of nitrate contamination in groundwater. ^31^

With regard to Computers & Industrial Engineering, another relevant journal that has made significant contributions to the field, a data mining-based framework for risk management in supply chains has been presented ^18^; in addition to a systematic review on the future of artificial intelligence and its impact on risk management in supply chains. ^33^

Similarly, journals such as Geomatics, Natural Hazards and Risk and Safety Science also stood out in terms of impact. A machine learning approach for flood risk assessment using remote sensing data and GIS was published in 2017. ^34^ In the field of mining safety, artificial intelligence was applied to assess gas risks in coal mines in 2921. ^35^

Finally, in the group of journals with high scientific productivity but low number of citations, IEEE Access and Sensors were found. Studies based on machine learning of the cryptocurrency market for financial risk management have been carried out ^36^; other investigations have studied the profiling of cybernetic attackers for risk analysis through machine learning. ^37^ These journals have been essential for the dissemination of innovative and relevant research in the field of machine learning applications in risk management.

In terms of the main countries, Figure 5 shows that South Korea, the United States and China stand out for their scientific production on the application of machine learning in risk management; these countries have made significant contributions in this area, demonstrating their leadership in the research and application of advanced machine learning techniques for risk management; In the case of South Korea, machine learning-based analyses have been conducted for financial risk management in the cryptocurrency market ^36^; similarly, in the field of sports medicine, machine learning models have been applied to analyze the risks of anterior cruciate ligament injuries. ^38^

Researchers in the United States worked on the development of risk assessment tools using machine learning; in 2023 they proposed an approach to integrate machine learning predictions into perioperative risk management ^6^ and in 2022 they developed machine learning models for individualized assessment of necrosis risk in mastectomy flaps. ^39^

In the same way, China is a benchmark in the application of automatic learning for risk management, studies have addressed risk assessment in supply chains using big data and machine learning, ^1^ in addition, they have proposed a machine learning-based method for pre-eclampsia risk assessment and related gene discovery. ^40^

Looking at Italy and India, although they are mainly recognized for their scientific productivity, they also have valuable research in risk management with the application of machine learning, in 2023 a study was conducted on defect detection using machine learning for asset risk management of existing bridges, ^41^ as well as in 2022 they focused on the use of deep learning and data analytics to improve agility in risk management in supply chains. ^23^

However, the leadership of the aforementioned countries is not solely attributable to the high number of publications; strategic factors such as innovation policies, financing, and the development of technological infrastructure are also contributing elements. In the case of China, the government has actively promoted artificial intelligence (AI) through the New Generation Artificial Intelligence Development Plan, which has driven research and the application of machine learning models in various sectors, including financial and environmental risk management. Conversely, South Korea has prioritized digitalization and automation in strategic sectors, including cybersecurity and data analysis in industry, supported by an innovation ecosystem that links universities, companies, and the government. Finally, the United States maintains its leadership thanks to strong investment from private and government institutions, as well as the presence of centers of excellence in artificial intelligence at universities such as MIT, Stanford, and Carnegie Mellon.

Consequently, these nations have not only produced a substantial volume of studies but also significantly advanced the applicability of Machine Learning in risk management, encompassing the detection of financial fraud and the prediction of natural disasters.

### Analysis of the thematic evolution of the application of machine learning in risk management

For the thematic evolution in Figure 6, it was found that the concept of stacking had a significant relevance in the first years of the application of machine learning in risk management. One of the relevant studies in this sense was “Credit risk analysis using a hybrid data mining model”. ^42^ This concept combines several machine learning models in order to improve the accuracy of predictions and allows to deal effectively with the assessment of credit risk. This methodology paved the way for the use of more advanced techniques in risk management and laid the foundations for the thematic evolution of the literature on the application of machine learning in risk management. Over the years, knowledge has expanded, and new approaches have been incorporated.

In the current state of the subject, key concepts that have received significant attention in recent literature have been identified, highlighting their relevance in different research areas, among which the concept of “suicide” has been the subject of study in 2019. It is approached from the perspective of suicide risk management in patients through the analysis of data from electronic health records, ^43^ which allows the development of clinical support strategies for decision-making in suicide prevention, demonstrating the usefulness and potential of machine learning techniques in the field of mental health.

In 2020, the concept of ‘Related Cardiac Dysfunction’ was highlighted, with an emphasis on the application of machine learning to assess the risk of cardiac dysfunction in patients undergoing cancer treatment. ^44^ The use of machine learning techniques has enabled a more accurate and personalized assessment of the risk of cardiac damage associated with cancer treatment, with a significant impact on the quality of life and survival of cancer patients.

By 2021, the focus will be on the concept of “Xgboost”, a machine learning technique that has gained popularity due to its ability to improve the accuracy of predictors. ^26^ This methodology has been applied in the context of cardiovascular risk and stroke, allowing a more effective and reliable assessment of risk by incorporating predictors based on carotid plaques and coronary angiography determination.

On the other hand, the year 2022 was strongly influenced by the concept of “Covid-19”, where the use of machine learning has stood out in the field of risk management related to the pandemic. ^19^ The development of approaches based on artificial intelligence has been essential to assess the risk of infection in virtual visits, which has been relevant in medicine and in the adoption of preventive measures in the fight against the spread of the virus.

Finally, in 2023, the concept of “urban trees” emerged as a topic of interest in risk management in urban areas, ^45^ the application of artificial intelligence has allowed the optimization of the associated risk assessment. to urban trees in localities in Brazil, facilitating the identification and prioritization of risk prevention and mitigation actions in urban environments.

### Analysis of thematic clusters on the application of machine learning in risk managemente

The bibliometric analysis enabled the identification of distinct thematic clusters, as illustrated in Figure 7, which highlight the affinities between the most recurrent terms in the scientific literature. One of the most prominent clusters, depicted in red, comprises keywords such as “Random Forest,” “Machine Learning Algorithm,” “Credit Risk Assessment,” “Support Vector Machine,” “Logistic Regression,” and “SVM.” This cluster underscores the strong association between machine learning techniques and credit risk assessment, emphasizing the specific algorithms widely applied in this domain. Another significant cluster, shown in dark green, includes terms like “credit risk,” “conjoint learning,” “feature selection,” “neural networks,” “clustering,” and “fuzzy logic.” This cluster reflects the integration of machine learning techniques with feature selection methods to enhance credit risk assessment. A notable study from 2021 exemplifies this approach by incorporating expert insight with genetic algorithms to optimize feature selection in credit risk evaluation. ^9^

A recently emerging cluster pertaining to digital assets underscores the mounting significance of optimizing portfolio management and risk assessment through the application of deep learning techniques. This notion is exemplified by research endeavors concentrating on predictive analysis, with the aim of enhancing decision-making processes within this rapidly evolving domain. ^46^ Beyond merely reflecting prevailing research trends, these clusters address specific challenges inherent to risk management. For instance, one cluster centers on “risk prediction,” “deep learning,” and “inference models,” signifying an escalating emphasis on enhancing the predictive accuracy of machine learning models. This aligns with real-world applications in banking and insurance, where deep learning has enhanced early fraud detection. ^47^ Another thematic cluster revolves around “cybersecurity,” “anomaly detection,” and “neural networks,” showcasing the role of machine learning in identifying and mitigating threats in digital environments. A notable instance is the implementation of anomaly detection models within corporate security systems, which have been instrumental in reducing cyberattack risks through proactive, AI-driven responses. ^46^

A third cluster, finally, encompasses “sustainability,” “environmental risk management,” and “data mining,” thereby illustrating the expanding role of machine learning in predicting natural disasters and optimizing mitigation strategies. Recent studies have demonstrated that analyzing large volumes of climate data with predictive models has improved responses to forest fires and extreme weather events. ^48^

Consequently, the co-occurrence analysis of keywords not only delineates contemporary research trends but also unveils nascent applications that are precipitating a transformation in risk management across diverse sectors.

### Analysis of frequency and conceptual validity around the use of machine learning in risk management

In the analysis of the Cartesian plane presented in Figure 8, quadrant 4 was identified as the one that contains decreasing or less used concepts compared to previous periods, in this quadrant are keywords such as “classification”, “logistic regression” and “decision tree”, these concepts, which in the past may have been more frequent in the scientific literature on the subject, show a decrease in their use in recent years.

For the concept of “classification”, relevant research was found that represents its past use, the authors applied a classification approach based on machine learning for the evaluation of spatiotemporal risks in crime data, ^49^ this concept may have been more prevalent in earlier studies related to data classification and analysis for risk management.

On the other hand, “logistic regression” has been used historically in financial risk management, as shown in, ^50^ but its presence seems to have decreased in recent studies on the topic. “Logistic regression" is a classic statistical method that has been widely used in various fields, but with the advancement of more complex machine learning techniques, its use in risk management may have decreased.

Finally, the concept of “Decision Tree”, which also shows a decreasing trend in recent years, has been applied in the optimization of risk analysis, as can be seen in, ^45^ where artificial intelligence is used for risk assessment in the context of managing trees in a specific region. While decision trees have been a valuable tool in risk management, other, more advanced approaches have gained popularity in recent years.

Quadrant 2 of the analysis of the Cartesian plane stood out for the grouping of emerging concepts in the scientific field of the application of machine learning in risk management; there are keywords such as “prediction”, “postpartum depression”, “Covid-19”, “big data” and “security”, which represent growing areas of great importance today and in the near future.

The concept of “prediction” has become fundamental in risk management, as it makes it possible to anticipate possible scenarios and assess the likelihood of future events, where ^49^ machine learning is used to perform conditional classification and assess spatio-temporal risks in crime data, the term “prediction” becomes relevant for informed decision-making and incident prevention.

For its part, ‘postpartum depression’ has also become a prominent research topic in risk management, as shown in, ^51^ where machine learning is applied to predict risk seeking. To help women with symptoms of postpartum depression, early detection and proper management of this condition is essential to minimize the risks associated with maternal mental health.

On the other hand, the “Covid-19” pandemic has led to a significant increase in risk studies and crisis management, as evidenced in, ^19^ where the issue of infection risk assessment using intelligence is addressed. During the virtual visit, the application of machine learning in the management of health-related risks was crucial to make informed decisions in the midst of a health crisis. Furthermore, the incorporation of machine learning into the healthcare sector has led to significant advancements in the realm of infection risk prediction and the optimization of resource allocation during health crises. These developments have not only led to improvements in patient outcomes but also facilitated the development of data-driven strategies for enhancing future pandemic preparedness. ^52^

“Big data” has also gained importance in risk management, as shown in, ^1^ where a method based on massive data is proposed for risk assessment in supply chains. The ability to process and analyze large amounts of data has significantly improved decision making and the identification of potential risks in complex environments.

Finally, the term ‘safety’ highlights the concern for safety in various sectors, and in Ref. 13, the use of machine learning for accident risk analysis is reviewed, particularly in Malaysia. This approach is relevant to industrial risk management and occupational safety, with the aim of preventing incidents and improving safety in the work environment.

Quadrant 1 of the Cartesian plane analysis revealed growing, leading and consolidated concepts in the application of machine learning to risk management. Among the prominent keywords in this quadrant are “prediction”, “big data”, “feature selection” and “deep learning”, which play a fundamental role today and have great potential for the near future.

“Prediction” is one of the most solid and widely studied concepts in risk management, it refers to the ability of machine learning to make accurate and reliable predictions about future events, research papers such as ^49^ show how the use of machine learning algorithms has significantly improved the ability to prevent risks in various areas, such as the analysis of crime data in spatiotemporal environments.

“Big data” has also established itself as an important concept for risk management in various fields, some research shows how the analysis of large amounts of data has allowed the identification of complex patterns and trends in risk assessment in supply chains. ^1^ The effective use of “big data” provides a deeper and more complete vision of potential risks and facilitates more informed decision making.

“Feature Selection” is another integrated tool in risk management, and it has been shown that the use of feature selection techniques allows the identification of key variables that influence risk prediction, thus improving the accuracy and efficiency of risk models. In the case of machine learning, ^53^ this ability to select the most relevant features has a significant impact on the early identification and mitigation of risks in a variety of applications.

Finally, ‘deep learning’ has emerged as a powerful and promising approach to risk management, showing how the use of U-series architectures has revolutionized image analysis in stroke risk assessment, ^54^ ‘deep learning’ enables a deeper representation of data, which has led to significant advances in risk analysis and prediction in various fields such as medicine.

### Comparison with other studies

A number of systematic reviews have examined the application of machine learning in risk assessment across various domains, underscoring both recent advancements and prevailing challenges. Hegde and Rokseth ^12^ present a comprehensive analysis of machine learning applications in engineering risk assessment, emphasizing its role in predictive maintenance, failure detection, and hazard identification. The review underscores the benefits of machine learning in enhancing risk prediction accuracy but also notes persistent challenges such as data quality issues, interpretability constraints, and the need for domain-specific adaptation. While the study provides a technical perspective on engineering applications, it does not offer a comprehensive discussion on how these challenges translate to other critical sectors like healthcare and finance. In contrast, the present study aims to provide a cross-sectoral analysis, identifying shared obstacles and proposing a more integrative approach to machine learning adoption in risk management across multiple industries.

Bias and interpretability persist as pivotal concerns in the implementation of machine learning for risk assessment, particularly in sensitive domains such as healthcare and finance. Suri et al. ^55^ and Singh et al. ^56^ examine the role of artificial intelligence in cardiovascular disease risk assessment, emphasizing the impact of biased training data on model reliability and fairness. These reviews underscore the challenge of ensuring equitable predictions, as biases in medical datasets can disproportionately affect certain demographic groups. Concurrently, Ahmed et al. ^11^ and Dewasiri et al. ^57^ focus on the financial sector, identifying algorithmic bias, regulatory constraints, and transparency issues as major limitations of AI-driven risk management in banking. While these studies provide domain-specific insights, they do not establish broader connections between these recurring challenges across industries. In contrast, our study makes a significant contribution by synthesizing findings from multiple sectors. It demonstrates how similar limitations—such as bias, regulatory concerns, and interpretability—hinder machine learning adoption in risk management universally.

A significant lacuna in extant reviews pertains to the absence of a structured research agenda to address unresolved challenges in machine learning-driven risk management. Dewasiri et al. ^57^ offer a discussion of the role of AI in banking and propose potential future directions; however, their review remains confined to financial applications. In a similar vein, Hegde and Rokseth ^12^ and Ahmed et al. ^11^ acknowledge the necessity for more robust regulatory frameworks and standardized evaluation metrics; however, they do not provide a roadmap for interdisciplinary solutions. Our study addresses this critical gap by developing a research agenda that spans multiple industries, outlining key areas for future exploration from 2007 to 2023. By integrating insights from existing systematic reviews and identifying persistent challenges across sectors, our work not only highlights research gaps but also provides actionable recommendations for improving the effectiveness and ethical implementation of machine learning in risk management.

### Classification of keywords on the use of machine learning in risk management according to their function

Table 1 is a fundamental component of this bibliometric study, as it classifies emerging and expanding machine learning concepts in risk management through an analysis of recent scientific literature. The key terms are organized according to function, thereby providing a systematic overview of the most active and promising research areas and applications in this evolving field.

**Table 1.** Classification of keywords according to their function. Own elaboration from Scopus and Web of Science.

Keyword	Related tools	Applications	Features
Prediction	Regression models, neural networks, support vector machines	Predicting future trends	Event prediction based on historical data.
Postpartum Depression	Natural language processing, sentiment analysis, classification	Identifying and monitoring postpartum depression	Language analysis to detect signs of depression.
Covid-19	Data mining, machine learning, epidemiological models	Predicting the spread of disease	Data analysis to understand the pandemic.
Big data	Data Analytics, Data Visualization, Data Integration	Extracting valuable information from large amounts of data	Managing and analyzing large data sets.
Safety	Risk Assessment, Fault Detection, Incident Reporting	Improve safety in the workplace	Early failure detection and accident prevention.
Feature selection	Recursive Feature Elimination, Principal Component Analysis	Selecting the most relevant features	Reducing dimensionality and improving model performance.
Deep Learning	Convolutional Neural Networks, Recurrent Neural Networks	Detect complex patterns	Hierarchical learning and high level representations.

The previous classification, as can be seen, is based on new concepts such as prediction, postpartum depression, Covid-19, big data, security, feature selection and deep learning, this classification becomes an important element for future research to support its studies based on these keywords.

### Practical implications

This bibliometric analysis revealed a thematic evolution from an initial focus on stacking to a deeper analysis of concepts such as Urban Trees, Covid-19, Xgboost, Related Cardiac Dysfunction and Suicide, which have important practical implications in the field of risk management. These results show that the scientific community is adapting its views to address urgent and emerging risks associated with specific problems, such as the Covid-19 pandemic and mental disorders.

Likewise, the analysis of the keyword co-occurrence network provides valuable information on the conceptual affinity of relevant terms in risk management, the identification of key terms such as Random Forest, Machine Learning Difference, Credit Risk Assessment, Support Vector Machine, Logistic Regression and Svm in the main thematic cluster suggests the importance of these tools in decision-making related to financial and credit risk management, this information can be very useful for professionals and experts in the field when designing strategies and models to reduce risk in different contexts.

In addition, keyword frequency and currency analysis show a different view of emerging trends in risk management: the fact that concepts such as classification, logistic regression and decision tree are declining in relevance, while terms such as prediction, postpartum depression, covid-19, big data and safety are emerging, reflects the changing dynamics of the field and the need to address new challenges and emerging issues.

On the other hand, the growth of concepts such as Prediction, Big Data, Feature Selection and Deep Learning highlights the importance of applying advanced machine learning techniques in risk management. These tools provide the ability to analyze large amounts of data, select key features and make accurate predictions, which can significantly improve decision making in risk identification, assessment and mitigation in different sectors and environments.

While an examination of publications on Machine Learning in risk management reveals a consistent growth in studies, it is imperative to assess the impact of these publications on the evolution of the field. S ignificant advancements have been identified in the application of deep learning models for risk prediction and analysis. These models facilitate more profound data representation, resulting in substantial progress in risk analysis and prediction across diverse domains, including medicine.

However, challenges persist in the applicability of these models, especially in critical areas such as explainability, fairness in algorithms, and regulatory concerns. The lack of interpretability of Machine Learning models hinders their adoption in environments where transparency is essential, such as financial management and decision-making in regulated sectors. Furthermore, fairness in risk prediction remains a challenge due to the potential presence of biases in training data.

The growth of deep learning in risk management is indicative of both an academic trend and significant advances in sectors such as finance and medicine. In the financial field, the implementation of deep neural networks has enabled improved fraud detection and credit assessment by analyzing large volumes of transactional data in real time. These models can identify complex patterns and risk signals more accurately than traditional methods, reducing financial losses and optimizing decision-making. In the healthcare sector, deep learning has enhanced medical risk assessment, facilitating more precise predictions regarding disease progression and patient response to various treatments.

Moreover, the emergence of sophisticated techniques such as causal inference and explainable artificial intelligence (XAI) has addressed a significant constraint of conventional models: the absence of interpretability. In the context of financial risk assessment, this development translates into the capacity to substantiate credit decisions to regulators and customers, thereby enhancing transparency and fostering trust in automated systems. In the domain of cybersecurity, the integration of deep learning with anomaly detection has led to the development of more robust systems capable of identifying attacks in real time, thereby safeguarding critical infrastructures and sensitive data. These advancements underscore the significance of the trends identified in bibliometrics, demonstrating their tangible impact on risk management and the continuous redefined mitigation strategies across multiple sectors.

### Limitations

Firstly, the selection of the databases used, such as Scopus and Web of Science, could have omitted some relevant publications in the field found in other sources not included in this study. This could have resulted in a partial view of the scientific production in the area studied.

Furthermore, while tools such as Microsoft Excel and VOSviewer have been employed to define bibliometric indicators of quantity, quality, and structure, these tools may not have fully captured the complexity and diversity of the scientific literature in this field. Some qualitative aspects of the publications, such as the depth of the analyses or the quality of the research methods used, may have been outside the scope of the quantitative indicators applied. Moreover, due to limitations in time and resources, the analysis might have excluded relevant publications, potentially affecting the representativeness and completeness of the results presented. While bibliometrics provides a robust quantitative analysis of scientific production in the field of machine learning and risk management, incorporating qualitative methodologies could enhance the interpretation of the findings. In subsequent studies, it is recommended that bibliometric analysis be complemented with expert interviews or case studies, which would facilitate a more comprehensive contextualization of the challenges and practical applications of these technologies in real-world settings.

The study emphasizes the importance of approaches such as deep learning and feature selection in risk management. However, it does not delve into the intricacies of emerging paradigms in the domain of machine learning. Among these, explainable artificial intelligence (XAI) emerges as a pivotal element, aiming to enhance the interpretability of models in risk management. Similarly, self-supervised learning and federated learning stand as promising methodologies to bolster the robustness and privacy of modeling. Finally, causal inference models have the potential to contribute to more informed decision-making in risk environments, representing a key area for future research. Despite the strides made in the implementation of machine learning algorithms in the domain of risk management, significant ethical and regulatory challenges persist. In sectors characterized by stringent regulations, such as finance and healthcare, the General Data Protection Regulation (GDPR) in Europe stipulates constraints on the utilization of personal data, mandating transparency and consent for the processing of information. In the context of financial institutions, for instance, regulations such as the GDPR mandate the development of models that are not only accurate but also comprehensible to auditors and clients. This necessity arises from the lack of interpretability in complex algorithms, such as deep neural networks, which hinders the acceptance of these models due to the difficulty in justifying critical decisions in risk contexts.

Additionally, the presence of bias and the absence of impartiality in machine learning models pose a significant challenge in the realm of risk management. In the healthcare sector, for instance, studies have demonstrated that certain models have the potential to perpetuate inequalities if the training data does not adequately reflect the diversity of the population, potentially leading to less accurate diagnoses in underrepresented groups. Similarly, in the financial sector, credit assessment algorithms can indirectly discriminate against certain customer profiles if they are based on biased historical patterns. To address these concerns, approaches such as explainable artificial intelligence (XAI) and algorithmic auditing have been developed. These methods aim to identify and correct biases in models.

However, implementing these approaches is challenging because it necessitates balancing model accuracy with transparency and fairness in its application. Furthermore, a crucial limitation that needs further discussion is the availability, quality, and potential bias of data in machine learning-based risk assessments. Many machine learning models are trained on large-scale datasets; however, these datasets frequently contain biases, inconsistencies, or incomplete information, which can result in inaccurate predictions. In healthcare, for instance, models trained on unrepresentative datasets may lack the capacity to generalize to broader populations, thereby reducing their effectiveness in real-world applications. Addressing these issues necessitates the implementation of more comprehensive data collection strategies, the employment of bias mitigation techniques, and the establishment of rigorous validation protocols. Exploration into adapting machine learning approaches used in the context of the COVD-19 pandemic to other infectious diseases and future health crises is warranted.

### Research gaps

Table 2 is attached, which presents the main research gaps identified in this topic. This table objectively and neutrally describes the areas where more attention and development are needed to close the existing gaps in the effective use of machine learning in risk management.

**Table 2.** Research gaps. Own elaboration from Scopus and Web of Science.

Gap category	Gaps identified	Justification	Questions for future researchers
Thematic gaps	- Lack of studies that specifically address the application of machine learning to natural disaster risk management.	Current bibliometrics show extensive research in the field of risk management, but few studies focus on the use of machine learning for natural disasters, which could be of great benefit.	How can machine learning improve the prediction and mitigation of risks associated with natural disasters such as earthquakes, floods or hurricanes? What are the best practices for applying machine learning in this context?
	- Lack of focus on the application of machine learning for risk management in specific industries, such as aviation or energy.	Although progress has been made in general risk management, there is little research on how machine learning could be adapted and optimized for specific industries.	What are the unique challenges of risk management in specific industries and how can machine learning effectively address them? What are the opportunities for implementing machine learning models in the aviation or energy industries?
Geographical gaps	- Insufficient representation of studies from developing countries in the application of machine learning for risk management.	Most research focuses on developed countries, which limits the understanding of how machine learning can benefit developing countries.	What are the specific barriers to the adoption of machine learning in developing countries in terms of resources, technology and training? How can these barriers be overcome to encourage wider use of machine learning in risk management in these regions?
	- Lack of studies addressing specific risks related to climate change and their application with machine learning in vulnerable regions.	Given the increasing risks associated with climate change, it is crucial to explore how machine learning can contribute to mitigation and adaptation in vulnerable regions.	How can machine learning improve climate risk assessment and forecasting in regions prone to extreme events? What specific machine learning approaches are most effective in addressing climate change-related risks?
Interdisciplinary gaps	- Limited integration of multidisciplinary approaches in the application of machine learning for risk management.	Bibliometrics show a predominance of other research focused on computer science, which may miss opportunities for collaboration with other disciplines.	How can collaboration between machine learning experts, risk management experts and other disciplines (such as engineering, social sciences or ecology) enrich the development and implementation of machine learning solutions for risk management?
	- Lack of research that combines uncertainty analysis in the application of machine learning to risk management.	Uncertainty assessment is crucial for informed decision-making in risk management, and its integration with machine learning could improve the accuracy and reliability of results.	How can uncertainty analysis be incorporated into machine learning models used for risk management? What are the best strategies for quantifying and communicating uncertainty in machine learning-based decision making?
Temporary gaps	- Limited availability of research that assesses the long-term sustainability of machine learning solutions for risk management.	It is important to understand how machine learning applications evolve and are maintained over time, as risks and challenges may change over time.	What is the long-term impact of machine learning solutions on risk management? How can organizations ensure that machine learning applications are sustainable and remain relevant in an ever-changing environment?

In terms of coherence, a total of 4 research gaps have been characterized in terms of subject matter, geography, interdisciplinarity and temporality, which provide information on the various ways in which future studies can be considered to meet existing research and knowledge needs.

### Research agenda

**Figure 9.**
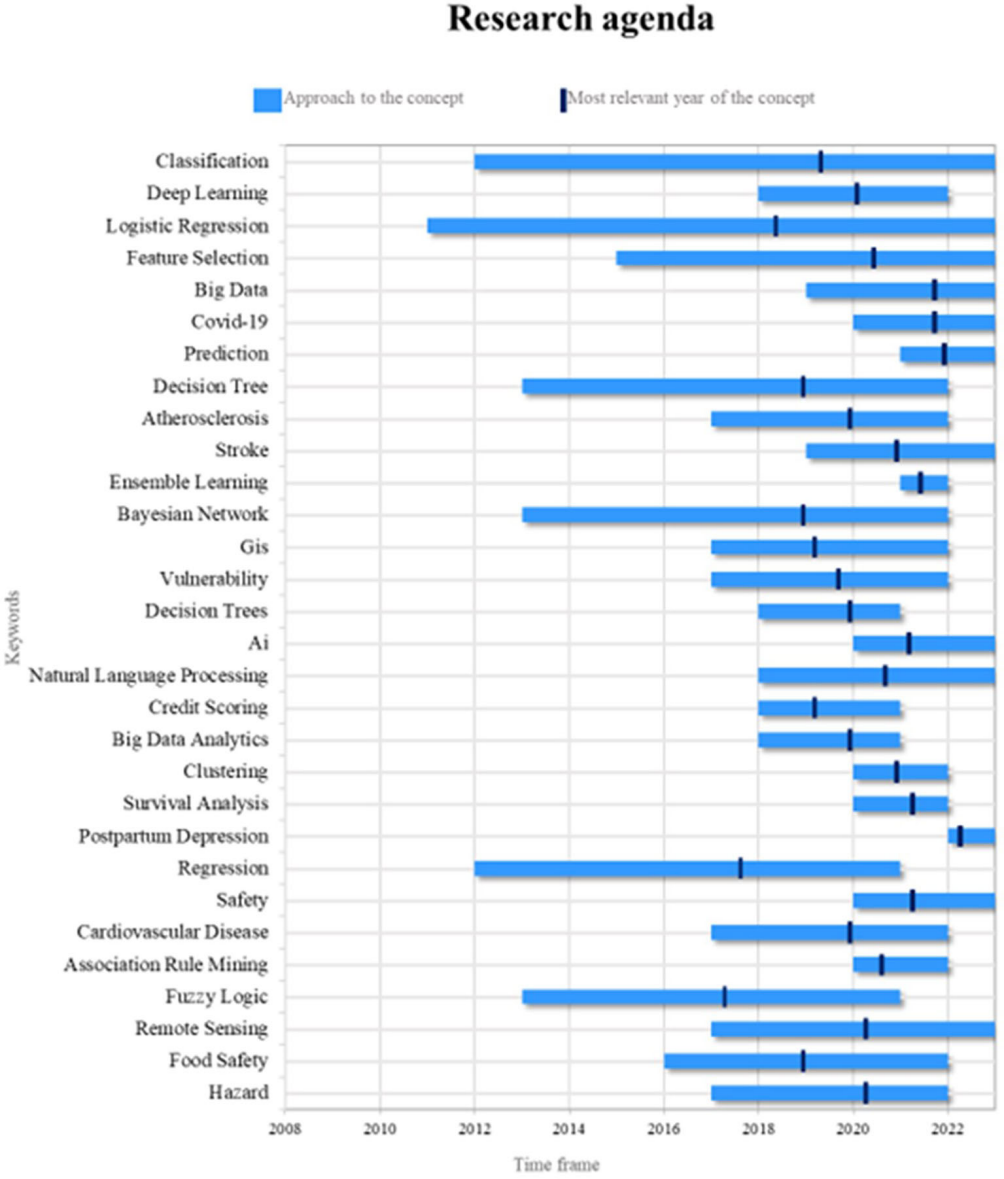
Research agenda. Own elaboration from Scopus and Web of Science.

Lastly, Figure 9 outlines a suggested research roadmap for this bibliometric analysis, aiming to provide a reference for other scholars conducting future scientific investigations on stereotypes categorized as current, emerging, and trending. To achieve this, two key aspects are analyzed: (1) the period during which the term has appeared in the literature and (2) the year of highest significance regarding scientific output, the latter highlighting the timeframe when the term played a more prominent role in academic work and was also examined in the most recent year.

Classification constitutes a foundational element in the implementation of machine learning in the context of risk management, as it facilitates the allocation of elements to predefined categories based on their characteristics. Classification is extensively employed to identify patterns of risk behavior and predict outcomes across diverse domains, ranging from credit analysis to fraud detection. Future research endeavors could encompass the exploration of more advanced classification methodologies, such as deep learning, with the objective of enhancing the precision and efficiency of risk models. Additionally, the integration of classification with other machine learning techniques, including feature selection and logistic regression, necessitates further investigation.

Logistic regression represents a valuable instrument in the realm of machine learning-based risk management, as it facilitates the estimation of event occurrence probabilities, thereby supporting risk assessment and informed decision-making processes. Future research endeavors could concentrate on enhancing the efficacy of logistic regression models through the incorporation of techniques such as regularization and hyperparameter optimization, with the objective of enhancing accuracy and robustness. Additionally, the exploration of its application in particular risk contexts—including early disease detection or financial loss prediction—and its integration with other machine learning techniques could further augment its utility.

In the contemporary context, prediction has emerged as a pivotal component of risk management, given its capacity to anticipate future occurrences. This concept has found extensive application in diverse domains, including climate risk estimation, market trend analysis, and health crisis prediction. Future studies should explore the potential of deep learning and big data techniques to enhance model accuracy and predictive capability. Additionally, research endeavors should prioritize the application of predictive models to emerging risks, such as those associated with the ongoing pandemic of SARS-CoV-2, also known as “covid-19,” and ensure their adaptability to dynamic and uncertain environments.

Machine learning has played a critical role in managing risks associated with the virus, aiding in predicting its spread, identifying at-risk groups, and analyzing epidemiological data. Future research could explore its contributions to early outbreak detection, vaccine strategy optimization, and public health decision-making. Furthermore, investigating how machine learning approaches used for the virus can be applied to other infectious diseases and future health crises is essential.

The increasing accessibility of substantial datasets has prompted the implementation of big data in risk management, particularly in the domains of information security and natural disaster risk assessment. Future research endeavors should explore the integration of big data with advanced machine learning techniques, such as deep learning, to optimize its capabilities. Moreover, addressing challenges related to large-scale data processing, including issues of data privacy and quality, is imperative to ensure the reliability and efficacy of risk management.

In the contemporary context, security has emerged as a pivotal component of risk management, particularly in the domains of intrusion detection, occupational risk analysis, and transportation safety. The future direction of research could entail the investigation of how deep learning and neural networks can augment threat detection and adaptability. Moreover, machine learning has the potential to contribute to the mitigation of cybersecurity challenges, such as the development of defense systems and the protection of confidential data. Beyond these applications, this study underscores the existence of several research gaps that necessitate further investigation. While certain domains remain underexplored, it is essential to critically analyze these gaps and propose concrete research directions to advance machine learning-based risk management.

One key area for future research is the development of hybrid models that integrate machine learning techniques with traditional statistical risk models. While ML-based models offer superior predictive accuracy, conventional statistical methods provide interpretability and robustness. Hybrid approaches could leverage the strengths of both paradigms, improving predictive performance while ensuring transparency. This is particularly salient in highly regulated sectors such as finance and healthcare, where decisions must be both accurate and justifiable.

Another crucial avenue to explore is the advancement of interpretability techniques for risk management models. Despite the advancements in explainable artificial intelligence (XAI), challenges persist in making complex machine learning algorithms understandable to non-technical stakeholders, such as regulators, auditors, and decision-makers. Future research should prioritize the development of interpretability techniques tailored to risk management applications, ensuring compliance with regulatory requirements while maintaining predictive power.

The availability, quality, and potential bias of data in machine learning-based risk assessment is another critical concern. A significant number of machine learning models are found to be reliant on training datasets that are marred by bias, resulting in erroneous predictions that have the potential to perpetuate existing inequalities. It is imperative for future studies to concentrate on the development of robust data curation techniques, fairness-aware algorithms, and validation frameworks. These endeavors are of paramount importance in fields such as credit risk assessment and healthcare diagnostics, where the presence of biased models can have substantial ethical and social ramifications.

Furthermore, disparities in the adoption of machine learning (ML) across geographical regions necessitate additional scrutiny. The implementation of ML in risk management exhibits variations due to the influence of regulatory, economic, and infrastructural factors. Comparative studies could analyze these variables to comprehend global variations and identify best practices that are adaptable to diverse contexts. Such research could inform policy recommendations to promote equitable access to AI-driven risk management solutions, particularly in developing economies.

Finally, the expansion of ML applications to emerging risk domains presents significant opportunities for future research. As risks evolve across industries, machine learning models must continuously adapt to new threats, including climate-related financial risks, cybersecurity vulnerabilities, and global health crises. For example, ML approaches used during the pandemic could be repurposed for other infectious diseases and future health emergencies.

Consequently, investigating the adaptability and optimization of machine learning techniques for emerging risks is imperative to maintain the efficacy and relevance of AI-driven risk management in dynamic environments. Addressing these research gaps is pivotal to advancing the field of machine learning-based risk management towards more effective, transparent, and ethically sound implementations. Future studies should prioritize these directions to ensure that machine learning technologies are not only innovative but also aligned with practical, regulatory, and societal needs.

## Conclusion

According to the bibliometric analysis of publication frequency and validity, the years 2023, 2022, 2021 and 2020 were the most significant in terms of interest in the use of machine learning in risk management. This indicates a growing interest and understanding of the importance of using machine learning to address risk management challenges in various sectors. It also suggests that there has been a significant increase in recent years in scientific production on this topic.

The scientific literature on the use of machine learning concludes that for risk management it has also shown cubic exponential growth, reflecting a constantly expanding field of study. This pattern of growth indicates that the topic has gained relevance and acceptance within the scientific community and will likely remain a busy and productive area of research for the foreseeable future.

The authors Laird, Suri and Saba, who have made significant contributions to the development of the literature, serve as the main research references in this area. The journals Science of the Total Environment and Computers and Industrial Engineering were also cited as important sources of literature on the subject. China, South Korea and the United States are the top three producers of scientific research, demonstrating their commitment to studying the use of machine learning for risk management.

The thematic development of the literature has changed significantly; we can conclude that in the past it focused on stacking, but now it focuses more on issues such as urban trees and Covid-19. This indicates the need to continue to explore new application areas and to modify risk management tactics to address current issues.

The analysis of thematic clusters has identified a consolidated set of terms with a strong conceptual affinity. Examples include random forest, machine learning algorithm, credit risk assessment, support vector machine, logistic regression and SVM. These fundamental findings provided a solid framework for further study and identified areas that should be prioritized for risk management modelling and methodologies.

In addition to the above findings, it is concluded that bibliometrics show that emerging keywords such as prediction, postpartum depression, Covid-19, big data, and security reflect highly relevant topics in the use of machine learning in risk management. These new ideas represent an expanding field of research and present opportunities to address specific problems, such as predicting future events, managing public health risks during pandemics, and incorporating big data to improve decision making.

Meanwhile, analysis of popular search terms such as prediction, big data, feature selection and deep learning highlights the importance of ongoing research in these areas to enhance and advance machine learning applications in industry. Risk management. These developed and extended ideas are trends in the development of methodologies and techniques applied to the difficulties of risk management in different context.

## Ethics and consent

Ethical approval and Consent were not required.

## Data Availability

No data are associated with this article. Zenodo: Machine Learning Applications in Risk Management: Trends and Research Agenda. https://doi.org/10.5281/zenodo.14841885. ^58^ The project contains the following extended data:
1. Dataset.xlsm (Raw data supporting the findings of this study). 2. PRISMA Checklist.docx (Checklist detailing compliance with PRISMA 2020 guidelines). 3. PRISMA flowchart.jpg (PRISMA flowchart) The data and materials are publicly available under a Creative Commons Attribution 4.0 International (CC BY 4.0) license. PRISMA Checklist for Machine Learning Applications in Risk Management: Trends and Research Agenda. https://doi.org/10.5281/zenodo.14841885. ^58^
