# Supplementary material for: Machine learning applications in risk management: Trends and research agenda
Source: F1000Res. 2025 Apr 7;14:233. Originally published 2025 Feb 25. [Version 2] doi: 10.12688/f1000research.161993.2 (PMC12498517; doi:10.12688/f1000research.161993.2)
Supplement: Peer review history

# Reviewer report: Reviewer response for version 2

**DOI:** 10.5256/f1000research.179212.r376695

**Published:** 2025-05-09

**Authors:** Tian Tian

**Affiliations:** 1 Department of Applied Math, Illinois Institute of Technology College of Computing, Chicago, Illinois, USA

Reviewer Comments

 General Comments:

 This paper offers a thorough bibliometric analysis of machine learning (ML) applications to risk management across multiple sectors. It successfully identifies key models (e.g., Random Forest, SVM, Deep Learning) and emerging research themes (e.g., COVID-19 risk management, mental health prediction). The methodology is grounded in PRISMA-2020 and uses Scopus and Web of Science databases.

 The work is timely and essential; however, I have several suggestions to enhance its methodological rigor, clarity, and technical depth.

 Major Comments:

**1. Data Bias and Coverage**

- *Comment:* Exclusive reliance on Scopus and WoS could introduce selection bias, especially regarding non-English or regional publications.
- *Suggestion:* Explicitly acknowledge this in the Methods section and consider adding a table comparing database coverage. Discuss possible limitations.

**2. Keyword Normalization**

- *Comment:* The method for keyword standardization (e.g., "ML" vs. "machine learning") is unclear.
- *Suggestion:* Describe in the Methods section how synonyms were handled, whether via NLP (lemmatization) or manual curation. A supplementary glossary of merged terms would be valuable.

**3. Trend Quantification and Validation**

- *Comment:* The 98.99% growth rate could benefit from deeper statistical validation.
- *Suggestion:* Include a time-series analysis using Compound Annual Growth Rate (CAGR) or regression models. Visualize growth with confidence intervals.

**4. Distinction Between Emerging and Transient Trends**

- *Comment:* Emerging trends (e.g., COVID-19) are not distinguished from potentially transient topics.
- *Suggestion:* Apply methods like Kleinberg’s burst detection to validate long-term vs. short-term keyword trends.

**5. Technical Rigor: Mathematical Formalization**

- *Comment:* Key claims about feature selection and model improvement are not mathematically formalized.
- *Suggestion:* Add key equations in the Methods or Discussion, such as:

- Risk as a function of selected features and model output:

- Feature selection improves model performance:

- Risk prediction quality depends on data and model interpretability:

- Emerging risk prediction with new data:

Mathematical expression and explicitness for the method would enhance technical rigor and link mathematics to thematic clusters.

**6. Handling Multicollinearity in Keywords**

- *Comment:* Highly correlated keywords (e.g., "deep learning" and "neural networks") could distort the co-occurrence network.
- *Suggestion:* Clarify whether anti-correlation measures (e.g., PCA, VIF) were used. Adding a correlation matrix for top keywords would enhance transparency.

Is the work clearly and accurately presented and does it cite the current literature?

Partly

If applicable, is the statistical analysis and its interpretation appropriate?

Partly

Are all the source data underlying the results available to ensure full reproducibility?

No

Is the study design appropriate and is the work technically sound?

Partly

Are the conclusions drawn adequately supported by the results?

Partly

Are sufficient details of methods and analysis provided to allow replication by others?

No

Reviewer Expertise:

AI application on business analysis, industrial management,  Quant finance, fintech, management science, machine learning, and crypto quant research.

I confirm that I have read this submission and believe that I have an appropriate level of expertise to confirm that it is of an acceptable scientific standard, however I have significant reservations, as outlined above.

# Reviewer report: Reviewer response for version 2

**DOI:** 10.5256/f1000research.179212.r376534

**Published:** 2025-05-08

**Authors:** Rakibul Hasan Chowdhury

**Affiliations:** 1 University of Portsmouth, Portsmouth, UK

The revised version of the manuscript presents a significantly improved and well-structured contribution to the domain of machine learning (ML) in risk management. The authors have effectively addressed the earlier reviewer concerns and strengthened the analytical depth, methodological transparency, and contextual relevance of the study.

 Key Strengths:

- **Enhanced Abstract and Problem Statement:** The abstract now clearly reflects the study’s novelty and contribution, while the introduction presents a more focused articulation of gaps in current literature.
- **Improved Methodological Rigor:** The detailed justification of the bibliometric approach, alignment with PRISMA-2020 standards, and elaboration on inclusion/exclusion criteria reflect a robust and replicable research design.
- **Expanded Discussion on Emerging Trends:** The inclusion of emerging applications such as Explainable AI (XAI), causal inference, and self-supervised learning offers valuable foresight for future research directions.
- **Cross-sectoral Relevance:** The manuscript’s interdisciplinary coverage—from supply chain and healthcare to financial services and environmental safety—underscores its broad practical applicability.
- **Ethical and Regulatory Insight:** The discussion of data bias, model interpretability, and compliance challenges provides a thoughtful ethical framework, which is essential for deployment in high-stakes sectors.
- **Research Agenda:** The refined agenda in the final section is clear, actionable, and addresses sector-specific and global challenges, positioning the article as a foundational reference for future scholars in the field.

Minor Suggestions:

- Although the keyword clustering is informative, future iterations of this work could benefit from a more visual and interactive representation of conceptual linkages, such as dynamic dashboards or visual analytics tools.
- Consider consolidating the research gaps table with illustrative examples for each gap to enhance usability for policy-makers and academic practitioners alike.

Final Note:

 This is a timely and comprehensive study that contributes significantly to the literature on the evolving integration of ML in risk management. The authors' responsiveness to reviewer feedback and their commitment to scholarly rigor are commendable. I am pleased to approve this revised version for indexing.

Is the work clearly and accurately presented and does it cite the current literature?

Yes

If applicable, is the statistical analysis and its interpretation appropriate?

Yes

Are all the source data underlying the results available to ensure full reproducibility?

Yes

Is the study design appropriate and is the work technically sound?

Yes

Are the conclusions drawn adequately supported by the results?

Yes

Are sufficient details of methods and analysis provided to allow replication by others?

Yes

Reviewer Expertise:

Intelligent EnterpriseArtificial Intelligence (AI) in BusinessMachine Learning (ML) for Decision-MakingEnterprise Risk Management (ERM)Predictive Analytics in BusinessBig Data and Business IntelligenceAI-Driven Process OptimizationDigital Transformation in EnterprisesAutomated Decision Support SystemsStrategic AI Implementation in Organizations

I confirm that I have read this submission and believe that I have an appropriate level of expertise to confirm that it is of an acceptable scientific standard.

# Reviewer report: Reviewer response for version 2

**DOI:** 10.5256/f1000research.179212.r376696

**Published:** 2025-05-07

**Authors:** muskan Khan

**Affiliations:** 1 Karnatak University Dharwad, Dharwad, Karnataka, India

**Title:**

**Machine learning applications in risk management: Trends and research agenda**

**Authors:**

 Alejandro Valencia-Arias, Jesus Alberto Jimenez Garcia, Erica Agudelo-Ceballos, Aarón José Alberto Oré León, Ezequiel Martínez Rojas, Julio Leyrer Henríquez, Diana Marleny Ramírez-Ramírez

**Journal:**

 F1000Research (2025), Version 2

**1. Summary of the Study**

 This study presents a bibliometric analysis of the literature on machine learning (ML) applications in risk management, focusing on trends, thematic clusters, and research agendas from 2007 to 2023. It applies the PRISMA 2020 framework and analyzes literature from Scopus and Web of Science. Key contributions include identification of thematic trends (e.g., COVID-19, urban trees, credit risk), emerging methods (e.g., XAI, self-supervised learning), and gaps related to explainability, data bias, and regulatory issues.

**2. Strengths**

- **Comprehensive Coverage:** The paper uses a robust bibliometric approach, covering a 16-year period and evaluating trends using high-impact databases.
- **Methodological Rigor:** The PRISMA 2020 protocol adds credibility to the selection and filtering of studies.
- **Insightful Thematic Analysis:** Clear identification of research clusters and evolving keyword trends (e.g., deep learning, feature selection, COVID-19).
- **Critical Discussion:** The article evaluates technical and ethical challenges in ML for risk management, including transparency and fairness.
- **Actionable Research Agenda:** Provides a structured future roadmap and identifies thematic, geographic, and industrial research gaps.

**3. Weaknesses**

- **Overreliance on Bibliometrics:** While the quantitative analysis is strong, deeper qualitative insights (e.g., case studies or domain-specific applications) are limited.
- **Limited Industry-Specific Depth:** Although sectors like finance and healthcare are mentioned, the application discussions lack depth in domains like energy, aviation, or cybersecurity.
- **Redundancy in Content:** Some parts of the discussion and methodology are overly repetitive, especially in descriptions of keyword trends.
- **Figures and Tables:** The paper references several figures (e.g., keyword maps, thematic clusters), but their interpretability would benefit from improved visual clarity and tighter linkage to the narrative.

**4. Originality and Novelty**

 The study stands out for synthesizing cross-disciplinary ML applications in risk management and developing a multi-sector research agenda. The inclusion of emergent topics like explainable AI and postpartum depression in risk contexts enhances its novelty.

**5. Relevance and Impact**

 Given the increasing reliance on AI/ML in risk-centric domains, this study is timely and highly relevant. It will be useful for both academic researchers and industry practitioners aiming to align with emerging trends in digital risk mitigation.

**6. Recommendations for Improvement**

- Include more **sector-specific case studies** to contextualize the findings.
- Provide a **more detailed visual explanation** of the keyword co-occurrence and thematic evolution graphs.
- Reduce **repetition** in the discussion section to maintain reader engagement.
- Consider incorporating **qualitative validation**, such as expert interviews or applied examples, in future work.

**7. Final Recommendation**

**Approved with minor revisions**

 The paper presents a meaningful and methodologically sound contribution to the literature on machine learning in risk management. Addressing minor content refinement and clarity improvements will enhance its overall quality.

Is the work clearly and accurately presented and does it cite the current literature?

Yes

If applicable, is the statistical analysis and its interpretation appropriate?

Yes

Are all the source data underlying the results available to ensure full reproducibility?

Yes

Is the study design appropriate and is the work technically sound?

Yes

Are the conclusions drawn adequately supported by the results?

Yes

Are sufficient details of methods and analysis provided to allow replication by others?

Yes

Reviewer Expertise:

Machine Learning

I confirm that I have read this submission and believe that I have an appropriate level of expertise to confirm that it is of an acceptable scientific standard.

# Reviewer report: Reviewer response for version 2

**DOI:** 10.5256/f1000research.179212.r376533

**Published:** 2025-04-15

**Authors:** Ajay Vikram Singh

**Affiliations:** 1 German Federal Institute for Risk Assessment (BfR), Berlin, Germany

The revised manuscript has satisfactorily addressed all my comments and queries, and I recommend its acceptance.

Is the work clearly and accurately presented and does it cite the current literature?

Partly

If applicable, is the statistical analysis and its interpretation appropriate?

Partly

Are all the source data underlying the results available to ensure full reproducibility?

Partly

Is the study design appropriate and is the work technically sound?

Partly

Are the conclusions drawn adequately supported by the results?

Partly

Are sufficient details of methods and analysis provided to allow replication by others?

No

Reviewer Expertise:

Machine Learning, AI, QSAR PBPK, Computational Tox

I confirm that I have read this submission and believe that I have an appropriate level of expertise to confirm that it is of an acceptable scientific standard.

# Reviewer report: Reviewer response for version 1

**DOI:** 10.5256/f1000research.178111.r368692

**Published:** 2025-02-28

**Authors:** Rakibul Hasan Chowdhury

**Affiliations:** 1 University of Portsmouth, Portsmouth, UK

**Peer Review Report**

**Title:** *Machine Learning Applications in Risk Management: Trends and Research Agenda*

**Authors:** *Valencia-Arias A, Jimenez Garcia JA, Agudelo-Ceballos E et al.*

**Journal:** *F1000Research (2025, 14:233)*

**DOI:** *https://doi.org/10.12688/f1000research.161993.1*

**1. Summary of the Article**

 This study presents a **bibliometric analysis** on the application of **machine learning (ML) in risk management**, focusing on research trends, influential authors, top contributing countries, thematic clusters, and emerging keywords. The authors utilized the **PRISMA-2020** framework and retrieved data from **Scopus and Web of Science**.

**Key Findings:**

- **Growing Interest:** There has been a substantial increase in research publications on ML in risk management, particularly from 2020 to 2023, indicating the rising importance of this field.
- **Geographical Distribution:** The **United States, China, and South Korea** are leading contributors to research output.
- **Thematic Evolution:** The field has evolved from early topics like *stacking* to emerging applications in *urban trees*, *Covid-19 risk management*, and *XGBoost models*.
- **Methodological Advances:** Core ML techniques such as **Random Forest, SVM, Deep Learning, Feature Selection**, and **Big Data** have gained prominence.
- **Emerging Topics:** Keywords such as **prediction, postpartum depression, security, and big data** indicate new frontiers in ML-based risk management.

The authors also present a **research agenda**, highlighting areas for further exploration, including **cross-disciplinary ML integration, ethical considerations, and domain-specific risk modeling**.

**2. General Assessment**

 This article is **a well-structured bibliometric study** with a clear focus on the application of ML in risk management. The methodology follows established bibliometric practices, and the discussion provides **insightful interpretations** of trends. However, **some areas require improvement** to enhance scientific rigor, clarity, and completeness.

**3. Detailed Evaluation & Recommendations**

**3.1 Research Questions**

 The study sets out to answer several key research questions. Below is an evaluation of whether they are adequately addressed.

**Research Question** **Evaluation** **Comments & Suggestions for Improvement** **What are the years when there has been more interest in ML in risk management?** **Yes **Figure 2 effectively shows the growth trend.

**What is the growth rate of ML-related articles in risk management?** **Partly **The paper mentions exponential growth (98.99%), but a **statistical growth model** (e.g., CAGR) would be helpful.

**What are the main research references on ML in risk management?** **Yes **Identifies key authors (Laird, Suri, Li, etc.), but a **citation network analysis** could further enhance this.

**What is the thematic evolution derived from scientific production on ML in risk management?** **Yes **Uses keyword analysis to track shifts in focus.

**What are the main thematic clusters on ML in risk management?** **Yes **Figure 7 provides a clear keyword network.

**What are the growing and emerging keywords in ML-based risk management?** **Yes **Figure 8 identifies trending terms like *prediction* and *big data*.

**Which topics are key for designing a research agenda on ML in risk management?** **Yes **The research agenda is well-developed.

**Recommended Improvements:**

- **Growth Rate Analysis:** The article should include **Compound Annual Growth Rate (CAGR)** calculations instead of only stating a 98.99% increase.
- **Citation Network Analysis:** A **network visualization** of highly cited papers would provide a deeper understanding of research influence.

**3.2 Methodology**

**Strengths:**

 ✅ **PRISMA-2020 Framework:** The study adheres to PRISMA guidelines for systematic reviews, ensuring methodological rigor.

 ✅ **Database Selection:** Scopus & Web of Science are reputable sources for bibliometric research.

 ✅ **Search Strategy:** The search formulas are appropriate, targeting **risk management and ML-related keywords**.

**Areas for Improvement:**

 ❌ **Data Extraction & Preprocessing:**

- The paper does not discuss potential **data biases** (e.g., missing articles due to indexing limitations).
- The **data cleaning process** (e.g., removal of duplicates, handling of missing metadata) should be explicitly stated.

❌ **Quantitative Indicators:**

- The authors use **basic bibliometric measures (e.g., publication count, citation count)**.
- Advanced **scientometric techniques** like **h-index trends, impact factor analysis, and co-citation mapping** would strengthen the results.

**Suggested Enhancements:**

 ✔ **Clarify Data Cleaning Steps**: Describe how duplicate records, irrelevant studies, and indexing errors were managed.

 ✔ **Use Additional Bibliometric Indicators**: Include **h-index trends, impact factor, and author impact score** for deeper insights.

 ✔ **Consider Topic Modeling**: Using **Latent Dirichlet Allocation (LDA)** for automated topic extraction would provide an alternative view of thematic evolution.

**3.3 Results & Discussion**

**Strengths:**

 ✅ The findings are well-organized into **publication trends, author contributions, thematic clusters, and keyword evolution**.

 ✅ The figures (Figures 2–9) provide clear visualizations of key insights.

 ✅ The discussion effectively **connects bibliometric results with practical applications** in risk management.

**Areas for Improvement:**

 ❌ **Comparative Analysis Missing:**

- The study does not compare findings with **existing bibliometric reviews on ML in risk management**.
- A **benchmarking analysis** against similar studies could add more context.

❌ **Potential Bias in Geographical Analysis:**

- The study states that China, South Korea, and the U.S. dominate the field but **does not discuss potential biases**, such as language-based indexing preferences (English vs. non-English sources).

**Suggested Enhancements:**

 ✔ **Include a Literature Comparison Table:** Compare this study’s findings with prior bibliometric studies.

 ✔ **Discuss Possible Indexing Biases:** Consider how language limitations may influence the geographical distribution of publications.

**3.4 Research Agenda & Future Directions**

**Strengths:**

 ✅ The research agenda is **comprehensive and well-structured**.

 ✅ The paper identifies **four key research gaps**:

- **Lack of domain-specific ML adaptation**
- **Geographical disparities in ML applications**
- **Need for interdisciplinary risk models**
- **Integration of ML with traditional risk management frameworks**

**Areas for Improvement:**

 ❌ **Insufficient Ethical Considerations:**

- There is **no discussion of ethical implications**, such as ML bias, fairness, and explainability in risk management.

❌ **Regulatory & Industry Adoption Challenges Not Addressed:**

- The study does not explore **real-world adoption barriers**, such as **data privacy regulations (e.g., GDPR, HIPAA)** and **ML interpretability issues in high-stakes domains (e.g., finance, healthcare)**.

**Suggested Enhancements:**

 ✔ **Include a Section on ML Ethics:** Discuss potential **biases, transparency challenges, and fairness considerations**.

 ✔ **Explore Regulatory & Adoption Challenges:** Address **legal frameworks, industry regulations, and practical barriers to ML deployment** in risk management.

**4. Overall Recommendation & Final Comments**

**Criteria** **Rating (1-5)** **Comments** **Novelty & Relevance**⭐⭐⭐⭐⭐ (5)Timely and relevant topic, with practical significance.

**Methodological Rigor**⭐⭐⭐⭐ (4)Strong methodology but needs improved data cleaning and additional scientometric measures.

**Results & Discussion**⭐⭐⭐⭐ (4)Clear presentation but lacks comparative analysis.

**Future Research Agenda**⭐⭐⭐⭐ (4)Well-structured but should address ethical and regulatory challenges.

**Clarity & Writing Quality**⭐⭐⭐⭐ (4)Well-written but could be more concise in some sections.

**Final Verdict:**

 ✅ **Accept with Major Revisions**

 �� The article makes a **valuable contribution** but requires enhancements in **methodology, comparative analysis, and ethical discussions** to improve scientific rigor.

 Would you like me to draft an **author response letter** addressing these revisions?

Is the work clearly and accurately presented and does it cite the current literature?

Yes

If applicable, is the statistical analysis and its interpretation appropriate?

Yes

Are all the source data underlying the results available to ensure full reproducibility?

Yes

Is the study design appropriate and is the work technically sound?

Yes

Are the conclusions drawn adequately supported by the results?

Yes

Are sufficient details of methods and analysis provided to allow replication by others?

Yes

Reviewer Expertise:

Intelligent EnterpriseArtificial Intelligence (AI) in BusinessMachine Learning (ML) for Decision-MakingEnterprise Risk Management (ERM)Predictive Analytics in BusinessBig Data and Business IntelligenceAI-Driven Process OptimizationDigital Transformation in EnterprisesAutomated Decision Support SystemsStrategic AI Implementation in Organizations

I confirm that I have read this submission and believe that I have an appropriate level of expertise to confirm that it is of an acceptable scientific standard, however I have significant reservations, as outlined above.

# Reviewer report: Reviewer response for version 1

**DOI:** 10.5256/f1000research.178111.r368687

**Published:** 2025-02-28

**Authors:** Ajay Vikram Singh

**Affiliations:** 1 German Federal Institute for Risk Assessment (BfR), Berlin, Germany

The paper provides a valuable bibliometric analysis of machine learning applications in risk management, highlighting key trends, emerging topics, and leading contributors, reinforcing the growing importance of AI-driven approaches in risk assessment. However, the study requires **major revisions** to enhance **methodological transparency, critical discussion of real-world challenges, regulatory considerations, and actionable insights**, ensuring its findings contribute meaningfully to both academia and industry. My comments are given below-

**Abstract: **The abstract provides a broad overview of the field but does not clearly articulate the novel contributions of this study. It should explicitly state how the bibliometric analysis advances current knowledge beyond existing reviews in the field. The claim about "important research gaps" is vague. Specify which gaps exist and how this study addresses them with new findings. Instead of just stating that ML is growing in risk management, emphasize the emerging trends and their practical implications. Phrases like "growing interest in ML for risk management" or "cross-cutting nature of ML applications" should be replaced with quantifiable trends, e.g., "Between 2018 and 2023, ML-related publications in risk management increased by X%."

**Introduction**

**Need for a stronger problem statement**- The introduction does not effectively frame the specific research challenges in applying ML to risk management. A well-structured introduction should highlight why existing ML applications in risk management face limitations. Define specific obstacles such as interpretability, bias, regulatory concerns, and data limitations. Show how this study uniquely contributes to bridging these gaps

**Literature review lacks depth- **While some ML applications in risk management are mentioned (e.g., supply chain, healthcare, finance), there is no critical evaluation of previous works. The authors should incorporate a comparative analysis of existing systematic reviews on this topic to clarify what is missing and what this study adds.

**Ambiguous transition from traditional to AI-driven risk management**- The text should clarify how ML surpasses conventional risk assessment models, providing examples of cases where ML outperforms traditional methods.

**Methods**

 Bibliometric methodology lacks justification. The authors state they used the PRISMA 2020 framework but do not justify why this specific bibliometric method was chosen over others (e.g., systematic mapping studies). Also. With the sentence ´ “Logistic regression" is a classic statistical method that has been widely used in various fields, but with the advancement of more complex machine learning techniques, its use in risk management may have decreased, cite Chandrasekar V et al. [2025 (Ref-1)] https://doi.org/10.1016/j.jhazmat.2024.137071 to make bibliography up to date. Explain why the inclusion/exclusion criteria were structured this way and how they ensure robustness in capturing relevant literature. The search strategy in Scopus and Web of Science is mentioned, but no actual search queries or filters are provided. Authors should provide a supplementary table listing keywords, Boolean operators, and filters used. Bias assessment is superficial. The discussion on selection bias, publication bias, and potential missing studies should be expanded. The authors mention automated classifiers, but how were these validated? Overreliance on bibliometrics without qualitative insights. While bibliometric analysis is useful, the paper lacks in-depth discussions on real-world applications, challenges, and limitations of ML in risk management. A hybrid methodology combining bibliometrics with expert interviews or case studies would have strengthened this work.

**Results & Discussion**

**Limited discussion of emerging methodologies-** The study highlights deep learning and feature selection but fails to discuss newer paradigms, such as: Explainable AI (XAI) in risk management. Self-supervised learning and federated learning for improving ML robustness. Causal inference models for better decision-making in risk contexts.

**Overemphasis on publication trends without actionable insights**- The results section predominantly focuses on publication growth trends but lacks insights into what these trends mean for the field. Instead of just listing “most productive countries” or “most cited papers,” authors should: Identify key breakthroughs in ML for risk management over time. Highlight specific challenges (e.g., explainability, fairness, regulatory concerns) in high-impact ML applications. Cite Singh AV et al. [2024 (Ref-2)] https://doi.org/10.3389/ftox.2024.1461587 with the sentence ´deep learning’ enables a deeper representation of data, which has led to significant advances in risk analysis and prediction in various fields such as medicine´

**Country-specific trends should be contextualized**- The paper states that China, South Korea, and the U.S. lead ML research in risk management, but does not analyze why. A deeper dive into policy, funding, and institutional strengths in these countries would add significant value.

**Superficial discussion of thematic clusters**- The clustering analysis is presented without critical evaluation. Instead of just listing keyword groups, the authors should: Explain why certain themes emerged together. Provide real-world examples of these clusters (e.g., case studies where ML has successfully improved risk assessment).

**Weak connection between results and real-world impact**- The discussion does not sufficiently address how the identified trends translate into practical risk management applications. For example, how does the rise of deep learning address current gaps in financial or medical risk assessment?

**Lack of discussion on ethical and regulatory challenges- **Regulatory barriers to ML adoption in risk management (e.g., GDPR, explainability requirements in finance) should be included. Issues related to bias, fairness, and model interpretability in risk-sensitive areas (e.g., healthcare, finance) should be discussed in greater depth.

**Missing discussion on data limitations**- The paper does not consider the challenges of data availability, quality, and bias in ML-based risk assessments. Many ML models suffer from biased training data, leading to inaccurate predictions—this crucial issue is overlooked. With the sentence ´It is also important to explore how the machine learning approaches used for Covid -19 can be applied to other infectious diseases, and how they can be adapted to address future health crises´, cite Barghash RF et al. [2024 (Ref-3)] https://doi.org/10.3390/molecules29235564 a recent report to support the statement.

**Superficial treatment of research gaps-** The identified gaps (e.g., lack of studies in certain domains) are stated but not critically examined. The paper should propose concrete future research directions, such as: Developing hybrid models that combine ML with traditional statistical risk models. Improving interpretability techniques in ML risk models. Addressing geographical disparities in ML adoption for risk management.

Is the work clearly and accurately presented and does it cite the current literature?

Partly

If applicable, is the statistical analysis and its interpretation appropriate?

Partly

Are all the source data underlying the results available to ensure full reproducibility?

Partly

Is the study design appropriate and is the work technically sound?

Partly

Are the conclusions drawn adequately supported by the results?

Partly

Are sufficient details of methods and analysis provided to allow replication by others?

No

Reviewer Expertise:

Machine Learning, AI, QSAR PBPK, Computational Tox

I confirm that I have read this submission and believe that I have an appropriate level of expertise to state that I do not consider it to be of an acceptable scientific standard, for reasons outlined above.

## References

[1] : Quantitative prediction of toxicological points of departure using two-stage machine learning models: A new approach methodology (NAM) for chemical risk assessment. *J Hazard Mater* .2025;487: 10.1016/j.jhazmat.2024.137071 137071 10.1016/j.jhazmat.2024.137071 39808958

[2] : AI and ML-based risk assessment of chemicals: predicting carcinogenic risk from chemical-induced genomic instability. *Front Toxicol* .2024;6: 10.3389/ftox.2024.1461587 1461587 10.3389/ftox.2024.1461587 39659701 PMC11628524

[3] : Navigating the COVID-19 Therapeutic Landscape: Unveiling Novel Perspectives on FDA-Approved Medications, Vaccination Targets, and Emerging Novel Strategies. *Molecules* .2024;29(23) : 10.3390/molecules29235564 10.3390/molecules29235564 39683724 PMC11643501

# Response

**Published:** 2025-03-10

**Authors:** JHOANY ALEJANDRO VALENCIA ARIAS

Greetings. Thank you to the reviewers for their valuable corrections. The point-by-point response is attached below:

**Comment**: Abstract: The abstract provides a broad overview of the field but does not clearly articulate the novel contributions of this study. It should explicitly state how the bibliometric analysis advances current knowledge beyond existing reviews in the field. The claim about "important research gaps" is vague. Specify which gaps exist and how this study addresses them with new findings. Instead of just stating that ML is growing in risk management, emphasize the emerging trends and their practical implications. Phrases like "growing interest in ML for risk management" or "cross-cutting nature of ML applications" should be replaced with quantifiable trends, e.g., "Between 2018 and 2023, ML-related publications in risk management increased by X%."

**Response**: The abstract was improved, specifying gaps, emerging trends, and including quantifiable data as recommended.

**Comment:** Introduction

 Need for a stronger problem statement- The introduction does not effectively frame the specific research challenges in applying ML to risk management. A well-structured introduction should highlight why existing ML applications in risk management face limitations. Define specific obstacles such as interpretability, bias, regulatory concerns, and data limitations. Show how this study uniquely contributes to bridging these gaps

**Response: **The introduction was improved, adding limitations and an extra paragraph to strengthen the problem statement.

**Comment: **Literature review lacks depth- While some ML applications in risk management are mentioned (e.g., supply chain, healthcare, finance), there is no critical evaluation of previous works. The authors should incorporate a comparative analysis of existing systematic reviews on this topic to clarify what is missing and what this study adds.

**Response**: A subsection comparing prior studies was added to the discussion.

**Comment:** Ambiguous transition from traditional to AI-driven risk management- The text should clarify how ML surpasses conventional risk assessment models, providing examples of cases where ML outperforms traditional methods.

**Response: **A paragraph reinforcing the transition to AI-driven risk management was added.

**Comment**: Methods

 Bibliometric methodology lacks justification. The authors state they used the PRISMA 2020 framework but do not justify why this specific bibliometric method was chosen over others (e.g., systematic mapping studies). Also. With the sentence ´ “Logistic regression" is a classic statistical method that has been widely used in various fields, but with the advancement of more complex machine learning techniques, its use in risk management may have decreased, cite Chandrasekar V et al. [2025 (Ref-1)] https://doi.org/10.1016/j.jhazmat.2024.137071 to make bibliography up to date. Explain why the inclusion/exclusion criteria were structured this way and how they ensure robustness in capturing relevant literature. The search strategy in Scopus and Web of Science is mentioned, but no actual search queries or filters are provided. Authors should provide a supplementary table listing keywords, Boolean operators, and filters used. Bias assessment is superficial. The discussion on selection bias, publication bias, and potential missing studies should be expanded. The authors mention automated classifiers, but how were these validated? Overreliance on bibliometrics without qualitative insights. While bibliometric analysis is useful, the paper lacks in-depth discussions on real-world applications, challenges, and limitations of ML in risk management. A hybrid methodology combining bibliometrics with expert interviews or case studies would have strengthened this work.

**Response**: Methodology was improved, adding a limitation on the lack of qualitative methods.

**Comment:** Results & Discussion

 Limited discussion of emerging methodologies- The study highlights deep learning and feature selection but fails to discuss newer paradigms, such as: Explainable AI (XAI) in risk management. Self-supervised learning and federated learning for improving ML robustness. Causal inference models for better decision-making in risk contexts.

**Response**: A limitation on emerging methodologies was included.

**Comment**: Overemphasis on publication trends without actionable insights- The results section predominantly focuses on publication growth trends but lacks insights into what these trends mean for the field. Instead of just listing “most productive countries” or “most cited papers,” authors should: Identify key breakthroughs in ML for risk management over time. Highlight specific challenges (e.g., explainability, fairness, regulatory concerns) in high-impact ML applications. Cite Singh AV et al. [2024 (Ref-2)] https://doi.org/10.3389/ftox.2024.1461587 with the sentence ´deep learning’ enables a deeper representation of data, which has led to significant advances in risk analysis and prediction in various fields such as medicine´

**Response**: Implications were added to the discussion, and the recommended reference was included.

**Comment**: Country-specific trends should be contextualized- The paper states that China, South Korea, and the U.S. lead ML research in risk management, but does not analyze why. A deeper dive into policy, funding, and institutional strengths in these countries would add significant value.

**Response**: Country-specific analysis was expanded as recommended.

**Comment**: Superficial discussion of thematic clusters- The clustering analysis is presented without critical evaluation. Instead of just listing keyword groups, the authors should: Explain why certain themes emerged together. Provide real-world examples of these clusters (e.g., case studies where ML has successfully improved risk assessment).

**Response**: The keyword co-occurrence section was improved.

**Comment**: Weak connection between results and real-world impact- The discussion does not sufficiently address how the identified trends translate into practical risk management applications. For example, how does the rise of deep learning address current gaps in financial or medical risk assessment?

**Response**: Practical implications were added.

**Comment:** Lack of discussion on ethical and regulatory challenges- Regulatory barriers to ML adoption in risk management (e.g., GDPR, explainability requirements in finance) should be included. Issues related to bias, fairness, and model interpretability in risk-sensitive areas (e.g., healthcare, finance) should be discussed in greater depth.

**Response**: Limitations on ethical and regulatory challenges were added.

**Comment**: Missing discussion on data limitations- The paper does not consider the challenges of data availability, quality, and bias in ML-based risk assessments. Many ML models suffer from biased training data, leading to inaccurate predictions—this crucial issue is overlooked. With the sentence ´It is also important to explore how the machine learning approaches used for Covid -19 can be applied to other infectious diseases, and how they can be adapted to address future health crises´, cite Barghash RF et al. [2024 (Ref-3)] https://doi.org/10.3390/molecules29235564 a recent report to support the statement.

**Response**: Limitations were revised, and the recommended reference was included.

**Comment:** Superficial treatment of research gaps- The identified gaps (e.g., lack of studies in certain domains) are stated but not critically examined. The paper should propose concrete future research directions, such as: Developing hybrid models that combine ML with traditional statistical risk models. Improving interpretability techniques in ML risk models. Addressing geographical disparities in ML adoption for risk management.

**Response: **The future research agenda was enhanced as recommended.
